# New insights into Müllerian duct differentiation provided by single cell transcriptomics in the chicken embryo

**DOI:** 10.1371/journal.pgen.1012302

**Published:** 2026-10-09

**Authors:** Juan Lan Tan, Adele Barugahare, Andrew Thomas Major, Craig Allen Smith

**Affiliations:** 1 Department of Anatomy and Developmental Biology, Monash Biomedicine Discovery Institute, Monash University, Clayton, Australia; 2 Monash Genomics and Bioinformatics Platform, Monash University, Clayton, Australia; 3 School of Bioscience, Faculty of Science, The University of Melbourne, Melbourne, Australia; Geisel School of Medicine at Dartmouth, UNITED STATES OF AMERICA

## Abstract

In vertebrate embryos, Müllerian ducts give rise to the female reproductive tract. Here, we present a comprehensive analysis of Müllerian duct differentiation using single cell transcriptomics in the chicken embryo, a unique model for duct development. The data reveal greater cellular complexity of Müllerian duct differentiation than previously understood. Transcriptomic profiling of early formed Müllerian ducts identifies molecular signatures for the Müllerian duct Epithelium (MDE) and the Müllerian Duct Mesenchyme (MDM), the two principle compartments of the Müllerian duct. The MDE expresses *PAX2, LHX1, WNT7, LGR5, PDGFA/D*, and *CDH1/2*. The MDM is marked by *WNT4, EMX2, POSTN, OSR1, VIM,DMRT* and the EMT markers *ZEB1/2*. In the male, the regressing mesenchyme (MDM) has a unique molecular signature, expressing the transcription factors *MSX1, MSX2, RUNX2, GATA5, DLX3* and *DLX5*, and the signaling regulators *DKK4* and *NRG1* (Neuregulin). Single cell transcript profiling of the regressing male mesenchyme identifies new genes implicated in duct regression, including the WNT antagonist, DKK4. DKK4, responds to Anti-Mullerian Hormone induction and can induce duct regression when ectopically expressed in females. The duct mesenchyme in the female follows a different fate, with a molecular signature comprising *SMARCA2/ HMGA2/ZEB1/2/TWIST1/2/DMRT2* and persistent expression of *FOXE1, OSR2* and *HGF* (Hepatocyte Growth Factor). Cell clusters in the developing female ducts reflects spatial pre-patterning of the duct prior to later regionalisation. In contrast to AMH-mediated bilateral Müllerian duct regression in males, female chicken embryos show an unusual pattern of left duct differentiation and contralateral right duct regression. We find that right female duct does not regress in the same manner as in males, being transcriptionally distinct and lacking a population of regressing mesenchyme, despite presumably being mediated by AMH, as in males.

## Author summary

In vertebrate embryos, Müllerian ducts give rise to the female reproductive tract. Aberrations in duct development cause reproductive disorders, many of which have an unknown etiology. Here, we present a comprehensive analysis of Müllerian duct differentiation using single cell transcriptomics in the chicken embryo, a unique model for duct development. The data reveal greater cellular complexity of Müllerian duct differentiation than previously understood. Duct mesenchyme has two transcriptionally distinct populations, while duct epithelium shows distinct regionalised compartments. Trajectory analysis shows that these populations have different fates. Single cell transcript profiling of the regressing male mesenchyme identifies new genes implicated in duct regression. One of these male-restricted transcripts, *DKK4*, responds to Anti-Mullerian Hormone induction and can induce duct regression when ectopically expressed in females. Lastly, the right female duct does not regress in the same manner as in males, being transcriptionally distinct. These data provide new insights into Müllerian duct differentiation.

## Introduction

Proper development of the female reproductive tract is critical to sexual reproduction. The female reproductive tract of amniotic vertebrates (reptiles, birds and mammals) derives from a pair of embryonic tubes called the Müllerian ducts [1,2]. Paired Müllerian ducts initially form in both female and male embryos, in close association with the mesonephric kidneys and gonads. Once formed, the fate of the duct diverges dramatically between the sexes [3–11]. In male embryos, Müllerian ducts regress under the influence of testis-derived Anti-Müllerian Hormone (AMH) [3,12,13]. In mammalian female embryos, the absence of AMH enables the ducts to differentiate into the Fallopian tubes, uterus and upper one-third of the vagina [5,11,14,15]. Perturbations of Müllerian duct development have significant consequences for reproductive health. In human females, the most common Müllerian duct disorder is Mayer-Rokintansky-Kuster-Hauser syndrome (MRKH), which affects 1 in 4500 women [16–20]. In males, Persistent Müllerian Duct Syndrome occurs when the Müllerian ducts fail to regress. Individuals with PMDS have a 46,XY karyotype and typical male external genitalia. However, they also possess a uterus and Fallopian tubes and may have testicular descent abnormalities [21,22]. Most affected individuals have mutations in *AMH* or its receptor *AMHR2* [23,24]. However, 12% of cases have unknown molecular etiology, indicating the presence of novel genes that regulate male duct regression in addition to AMH [21,25]. Overall, most anomalies of the female reproductive tract cannot be explained at the genetic level, pointing to a need for a deeper knowledge of Müllerian duct formation and sexual differentiation.

Our understanding of the genetic and cellular regulation of Müllerian duct development has largely derived from studies on mouse and chicken embryos, in which duct formation is well characterized [26–37]. In both models, the Müllerian duct arises from a thickened placode of coelomic epithelial cells at the cranial pole of the mesonephric kidney [5,28,36]. The placode invaginates to form a meso-epithelial tube that progresses caudally through cell proliferation and migration [38,39]. The coelomic epithelium also undergoes an epithelial-to-mesenchyme transition, giving rise to mesenchyme that surrounds this tube, as shown by GFP-labeling studies in chick and mouse [39,40]. The Müllerian ducts hence comprise of three cellular compartments, the “outer” Müllerian surface epithelium (MSE), underlying Müllerian duct mesenchyme (MDM), and the “inner” meso-epithelial Müllerian duct epithelium (MDE) [28,39,40]. Gene targeting and mis-expression studies show that duct formation in chick and mouse involves the transcription factors LHX1, PAX2 and EMX2, together with WNT signaling [31,33,36,40–49]. Sexual differentiation involves duct regression in males versus development of functional oviducts in females. This phase is not well characterised at the molecular and cellular levels, except for the role of AMH in duct regression. *Amh*-null male mice retain Müllerian duct-derived structures, while transgenic female mice that mis-express *Amh* lack Müllerian duct-derived structures [11,13,22,50–52]. Downstream of Amh, the canonical WNT signaling pathway is crucial for regression, and β-catenin is required for duct regression in the mouse [53]^.^ Beyond the role of AMH and its putative downstream targets, there is little information on the cell biology of Müllerian duct regression.

With the aim of shedding new light on duct development, the study described here provides the first comprehensive single cell transcriptomic analysis of Müllerian duct sexual differentiation. The chicken embryo is particularly suited to studying the Müllerian duct. As embryonic development occurs outside the maternal body, it can be readily manipulated through *in ovo* electroporation of gene overexpression or knock-down constructs [54]. Furthermore, the chicken embryo exhibits an unusual pattern of duct sexual differentiation. While males undergo bilateral duct regression as in mammals, the right female duct also regresses in chicken, concurrent with regression of the right gonad [55,56]. This is because the female embryonic gonad also produces AMH (unlike in mouse), albeit at lower levels than in males [57–59]. The present study provides the most detailed analysis of the cellular complexity and gene regulatory landscape of Müllerian duct sexual differentiation to date. Using single cell transcriptomics, this study sampled over 30,000 cells and profiled the Müllerian ducts of both sexes prior to, during and after the onset of sexual differentiation. The single-cell RNA sequencing data revealed greater cellular complexity in the Müllerian duct than previously thought, identifying 17 transcriptionally distinct cell clusters throughout development. A specific population of mesenchymal cells was identified in the regressing male Müllerian duct, enriched for novel candidate duct regression genes. These male enriched transcripts were absent from the right female regressing duct, indicating that the mechanism of duct regression differs between the sexes. Furthermore, a candidate gene implicated in male duct regression, *DKK4*, was functionally analysed. Overexpression of *AMH* activated ectopic *DKK4* expression and induced duct regression in female ducts, while *DKK4* overexpression in female ducts induced robust duct regression and loss of duct mesenchyme. These data shed new light on the cellular and molecular mechanisms of Müllerian duct differentiation in vertebrate embryos.

## Results

### Single cell RNA-sequencing identifies diverse male and female cell populations in the Müllerian duct

In the chicken embryo, undifferentiated Müllerian ducts form by embryonic day (E) 6.5 (Hamburger and Hamilton (HH) stage 30) [60]. Ducts are initially morphologically identical in both sexes comprising an outer surface epithelium (the MSE, derived from coelomic epithelium), underlying duct mesenchyme (MDM) and inner duct epithelium (MDE) (S1 Fig). Male ducts exhibit bilateral regression in a caudo-cranial direction from E8.0 / HH stage 29–30, while both female ducts continue to grow. Marked asymmetry becomes apparent in females after E9.5 / HH stage 34–35, when the right duct begins to regress, cranio-caudally (i.e., in the opposite direction to male regression). In contrast, the left female duct continues to grow (S1A Fig). To examine the cellular and molecular events that underpin Müllerian duct differentiation, entire ducts were dissected, dissociated into single cells, and processed for single-cell RNA sequencing through the 10X Chromium system (10X Genomics). Five samples were processed in triplicate, corresponding to key stages of duct sexual differentiation (Fig 1A and 1B). The E6.5 mixed sex sample represents formation of the undifferentiated Müllerian ducts in both sexes. The E8.5 female and male samples correspond to sexual differentiation of the ducts, in which the female ducts continue to develop while the male ducts undergo bilateral regression. The E11.0 female left and right ducts represented asymmetrical duct development, in which the left duct continues to grow and the right duct regresses. Three technical replicates were analysed, and each replicate consisted of at least 3 individual ducts or duct pairs. After quality filtering, a total of 39,431 cells were sequenced across all samples. 5510 mean reads were counted per cell, and a median of 1007 genes were detected per cell. Of all sequenced cells, 30704 cells (88%) were female, and 8727 cells (22%) were male. The E6.5 mixed sex sample comprised 3314 cells (8% of total sequenced cells), of which 1872 cells (56% of E6.5 sample) were female and 1442 cells (44% of E6.5 sample) were male. The E8.5 female sample comprised of 9548 cells (24%), the E8.5 male sample comprised 7285 cells (18% of total sequenced cells), the E11.0 female left duct sample comprised of 11686 cells (30% of total sequenced cells) and the E11.0 female duct sample comprised of 7598 cells (19% of total sequenced cells). The dataset was visualised in a Uniform Manifold Approximation and Projection (UMAP) plot encompassing both sexes at different developmental stages. The cells were clustered based on transcriptional similarity at a Seurat resolution of 0.8. This identified 23 transcriptionally distinct clusters numbered 0–22. Cluster 0 contained the largest number of cells (4928 cells, 12%). Additionally, the largest five clusters contained 50% of the total number of sequenced cells, whereas the smallest seven clusters each represented less than 1% of the total number of cells (Cell barcodes, cluster information and UMAP coordinates provided in S1 Table).

**Fig 1:**
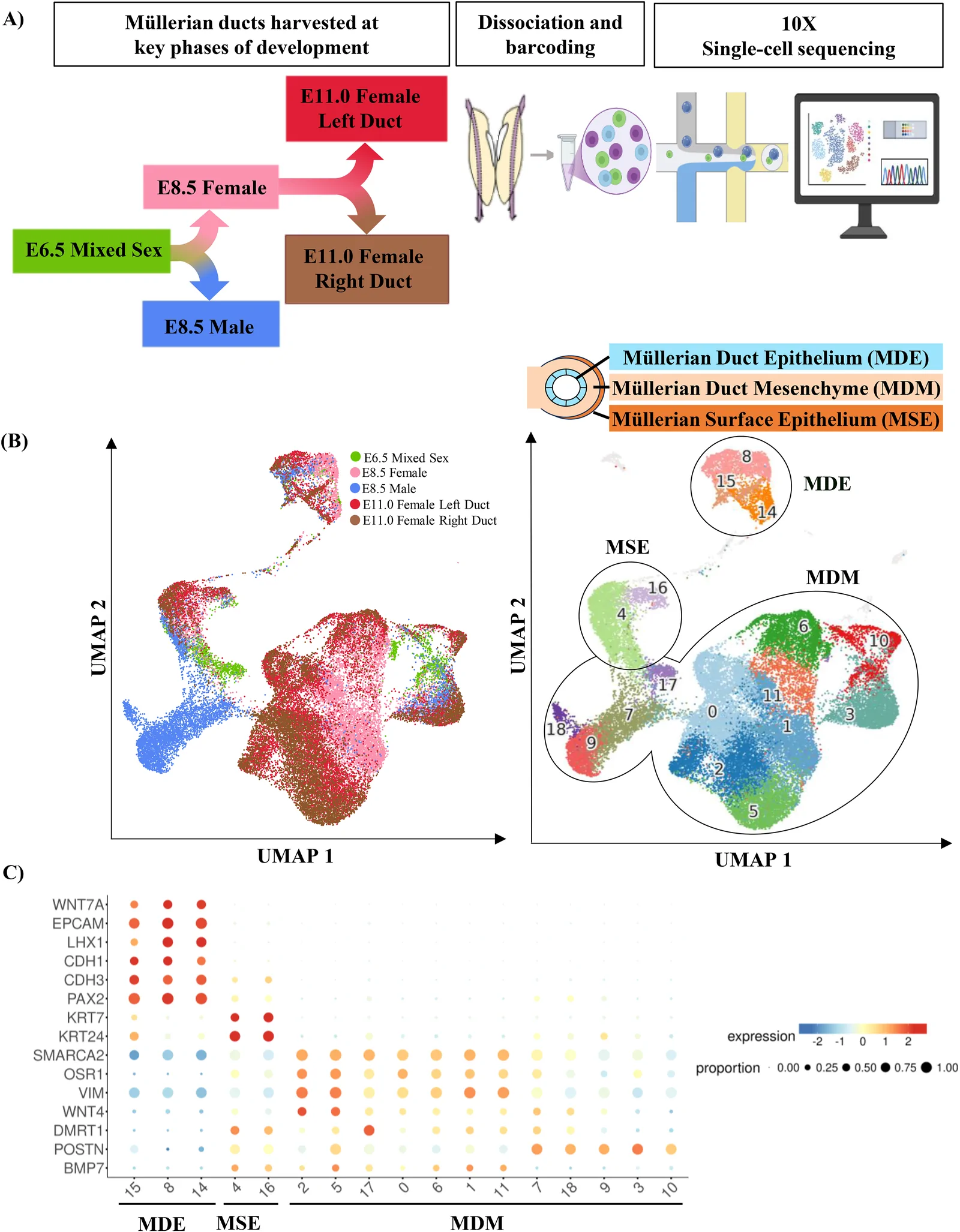
Single-cell sequencing identified 17 Müllerian duct clusters. **(A)** Five samples were processed for single-cell RNA sequencing, corresponding to key stages of duct differentiation. **(B)** Müllerian duct cells were visualised in a uniform manifold approximation and projection (UMAP) plot, colour-coded by sample of origin (Left), and cluster number (Right). Müllerian duct compartments are outlined on the UMAP (Right). **(C)** Bubble-plot of known Müllerian duct compartment markers used to determine cluster identity. Created in BioRender. Major, **A.** (2026) https://BioRender.com/36i9apw.

### Transcriptomic profiling of Müllerian ducts identifies molecular signatures of each compartment and reveals unique cell populations

To characterize the cell populations present in the datasets, differential gene expression analysis was performed across the 23 clusters (The top 50 upregulated genes in each cluster are provided in S1 Table). Six clusters were identified as non-Müllerian duct cells (S2A and S2B Fig). These non-Müllerian duct cell clusters were excluded from subsequent analyses. The Müllerian ducts consist of three compartments in cross-section, the outer Müllerian Surface Epithelium (MSE; derived from coelomic epithelium), the underlying Müllerian duct mesenchyme (MDM), and the inner tube of Müllerian duct epithelium (MDE) (Fig 1B). These compartments have long been considered homogenous cell populations, however this study found 17 clusters of Müllerian duct cells present throughout duct development, suggesting greater cellular complexity than previously thought. Differential gene expression analysis was used to assign the cell clusters to the three Müllerian duct compartments (Fig 1B). A heatmap of the top differentially expressed genes in each cluster was generated (S2C Fig) A bubble-plot was also generated, showing the expression of known Müllerian duct compartment markers across all clusters (Fig 1C). Twelve clusters, Clusters 0, 1, 2, 3, 5, 6, 7, 9, 10, 11, 17 and 18, were assigned as MDM (mesenchyme), due to the high expression of the mesenchyme marker *VIM*, as well as extracellular matrix-related transcripts such as *POSTN,* and known duct mesenchymal markers such as *DMRT1* and *WNT4, SMARCA2, OSR1* [33,40]*.* Three clusters, C8, C14 and C15, were assigned as MDE (duct epithelium) due to strong expression of the known duct epithelial markers *WNT7a*, *PAX2 DACH1/2* and *LHX1 (LIM1)* [36,61]. As the MDE is meso-epithelial in nature, these cells also expressed some epithelial markers including the Cadherins *CDH1/3* and *EPCAM* (epithelial cell adhesion-related), and lacked others such as the keratins, *KRT7/24*. Lastly, two clusters, C4 and C16, were assigned as MSE (Müllerian Surface Epithelium), marked by strong expression of keratins such as *KRT7/KRT24*, characteristic of true epithelia (Fig 1C). The MSE also expressed known coelomic epithelial markers *BMP7*, *WNT4, DMRT1, POSTN,* and *PAX2* (the latter shared with the MDE) [36]. A cell phase analysis was performed to examine the differences in the proliferation rates among the Müllerian duct clusters, using 95 cell cycle marker genes ((S3A and S3B Fig). Proliferative clusters were identified in each Müllerian duct compartment. Clusters 6, 7, 14 and 18 were in the G2M phase, and Cluster 11 was in the S phase. These were distributed across the three duct compartments (S3A Fig). The remaining clusters were in the G1 phase, indicative of a quiescent profile (S3A Fig).

### Chicken Müllerian ducts are undifferentiated at E6.5

In the E6.5 tissues, the MSE, MDE, and MDM were already present, and female and male ducts appear morphologically identical (S1 Fig). To determine whether Müllerian duct cells were transcriptionally sexually undifferentiated at this stage of development, a co-expression plot of the female-specific marker *HINTW* and the universal “housekeeping gene” *GAPDH* was generated (S4A Fig). The *HINTW* gene is located on the W sex chromosome, and is robustly expressed in all female cells, but not male cells [62,63]. Cells expressing *HINTW* and *GAPDH* localised to the same clusters on the UMAP, and no sex-specific cluster was identified at E6.5 (S4A Fig). Furthermore, when we performed UMAP and clustering analysis using only E6.5 cells, there were no sex-specific clusters detected (S4B Fig). These data indicate that Müllerian ducts were not yet sexually dimorphic with regard to transcripts at E6.5.

### Development of the MSE (Müllerian surface epithelium)

At E6.5, the duct has already formed into three compartments - the MSE, MDE and MDM. The MSE derives from the coelomic epithelium [39,40], and expresses the cytokeratins *KRT7* and *KRT24*, transcription factors such as *WT1* and *PAX2,* and the signaling factor *WNT4* (Fig 1C). At E6.5 and throughout duct development, the MSE comprised of two sub-populations, Clusters 4 and 16. Both populations were transcriptionally similar and expressed the known MSE markers above, however Cluster 16 comprised of proliferating cells enriched for cell cycle and DNA replication markers. We conclude that this compartment essentially comprises one distinct epithelial cell type. Some novel MSE marker transcripts identified at E6.5 include the transcription factors *SOX6* (a HMG box developmental regulator), *BNC1* (a zinc finger protein implicated in epithelial differentiation), *MSC* (Musculin), the signaling factor *WNT16,* and cell structure molecules *PODXL* and *GJA1* (S4C and S4D Fig). SOX6 expression was validated by immunostaining (S4E Fig). This population remained largely transcriptionally homogenous throughout subsequent duct development, though expressing a suite of additional genes after E6.5, such as *HGF* (Hepatocyte Growth Factor).

Sexual differentiation of the chicken Müllerian ducts involved significant transcriptional changes in the MDE and the MDM, where we identified novel cell subtypes and identify the molecular signatures of these cell types, as detailed below.

### Development of the MDE (Müllerian duct epithelium)

The MDE expressed known markers such as *PAX2*, *LHX1*, *WNT7A*, and the cadherins, *CDH1, CDH2* and *CDH3* (Fig 1C). Here, we add to this molecular signature with the following signaling transcripts enriched in the E6.5 MDE: *LGR5* (a stem cell marker), *PDGFA* and *PDGFD* (Plate-Derived Growth Factors), *FGFR2, BMPR1B*, the transcription factor *SIX1*, and the epithelial adhesion factor, *EPCAM*.

At E6.5 and throughout duct development, the MDE comprised of three sub-populations of meso-epithelial cells, represented by Clusters 8, 14 and 15. Two main populations of epithelia, Cluster 8 and 15, were transcriptionally very similar at E6.5, while Cluster 14 represented a proliferating population (S3 Fig). There was no sexually dimorphic gene expression in the MDE at E6.5. However, by E8.5, there was marked sexual dimorphism in the MDE transcriptome (S6A Fig), corresponding with the onset of duct regression in males. Additionally, Clusters 8 and 15 became regionally distinct in developing female ducts, but were lost in males, concomitant with duct regression.

The regressing male MDE was enriched for gene transcripts that included the transcription factors *SIM1* and *RORB* (Retinoic acid signaling-related nuclear receptor), and signaling molecules *BMP3*, *BMPER* and the RNA-binding splicing factor, *RBFOX1.* These genes are candidate regulators or mediators of male MDE regression, as they were not enriched in females (S6A Fig). Instead, E8.5 female MDE was enriched for the transcription factors *DLX5*, *MSX2*, and *VENTX* and the signaling factors/mediators, *HGF* (Hepatocyte Growth Factor) and *SMAD9* (S6A Fig).

Additionally, in the E8.5 and E11.0 female, the two populations of MDE (Clusters 8 and 15) showed segmental gene expression along the anterior-posterior axis. Differential gene expression analysis found that Cluster 8 was marked by expression of the neurotrophic factor receptor, *GFRA1*, while Cluster 15 was marked by *MSX1/2* expression (S6B Fig). *In situ* hybridisation confirmed expression of these genes in the MDE of the female urogenital system throughout duct formation and sexual differentiation (E6.5, E8.5 and E10.5) (S6C Fig). At E6.5, E8.5 and E11.0, *GFRA1* expression was present along the length of the MDE excluding the posterior region. It was also expressed transiently in the MDM. Conversely, *MSX1/2* expression is present only in the posterior region of the MDE at E11.0 (S6C Fig).

### Development of the MDM (Müllerian duct mesenchyme)

Single cell transcriptomic data showed that the MDM comprises more than one mesenchymal cell type and that these cell types have distinct molecular signatures that diverge in males and females during duct differentiation. At E6.5, the MDM was already apparent and represented by Cluster 17, 3 and 10 (Fig 2A). Cluster 17 was uniquely enriched at E6.5, while Clusters 3 and 10 were present throughout duct development. Cluster 17 was determined to be undifferentiated mesenchyme, and differential gene expression analysis was performed to investigate the genes regulating early duct mesenchyme specification. This cluster expressed known duct mesenchyme markers in chicken or mouse, such as *WNT4*, *EMX2, POSTN, OSR1, VIM* and *DMRT1* [33,40] (S5A Fig). Novel transcription factors were also linked to this population, such as *RUNX1* and *FOXE1* (S5C Fig). Interestingly, this cluster also expressed novel markers associated with axonal guidance (*NELL2, CNTN4*, *ROBO1*) and vascular endothelial cell migration (*ENOX1*) (S5C Fig). Genes encoding cell adhesion and matrix proteins (*VCAN*, *CDH4*, *HAPLN1, LTBP1*) were also all highly expressed in Cluster 17.

**Fig 2:**
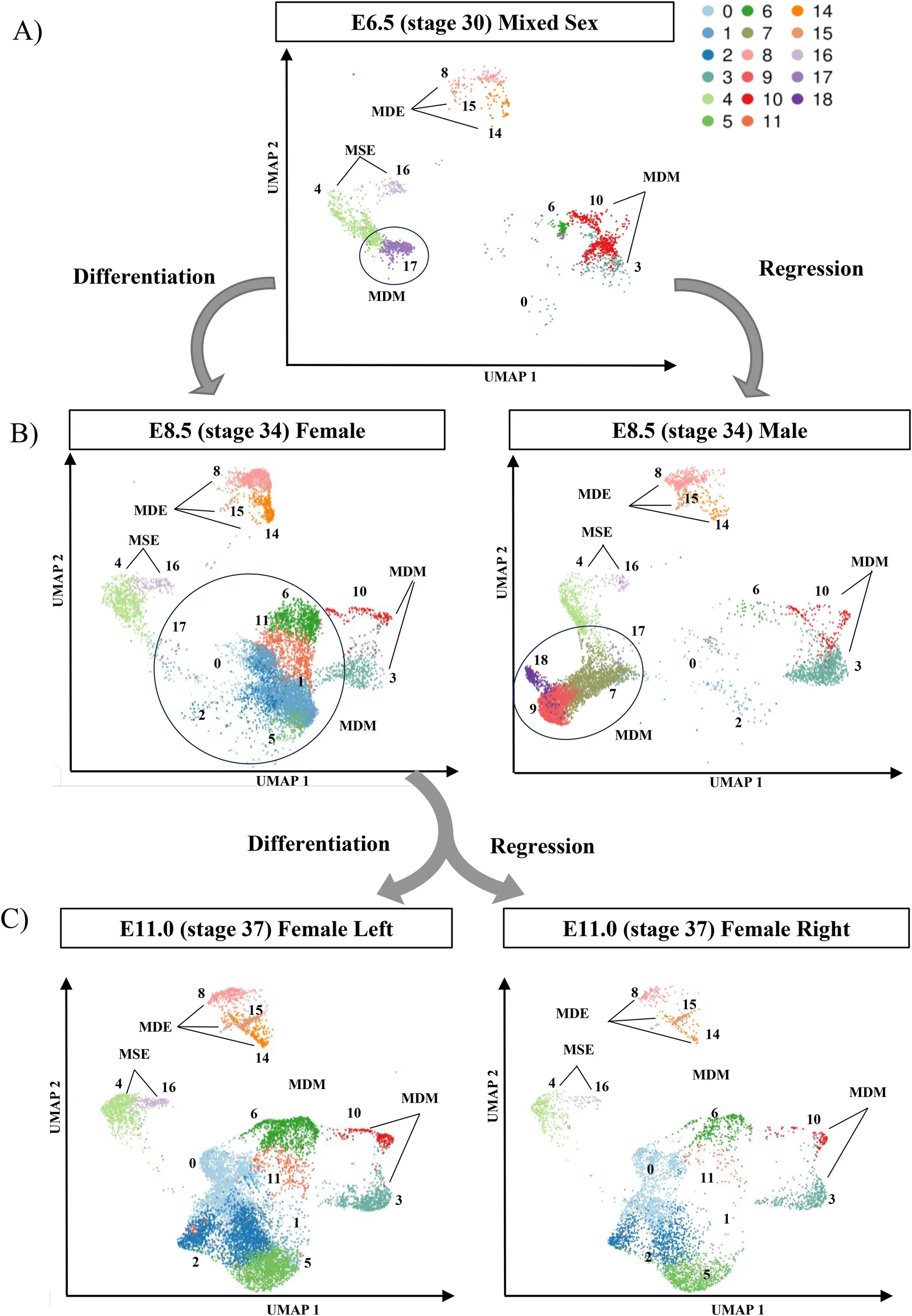
Tissue-specific UMAPs showing cell populations present at each stage of Müllerian duct differentiation. **A)** Eight cell clusters, indicated by the lines and circle, were present initially at E6.5. This included three MDE clusters (Clusters 8, 14 and 15), two CE clusters (Clusters 4 and 16), and three MDM clusters: Cluster 17 (circled) and two clusters (lines). **B)** By E8.5 (stage 34), the female MDM Cluster 17 had diversified into Clusters 1, 6, and 11 (circle). By E8.5 (stage 34), the male mesenchyme (MDM Cluster 17) had diversified into Clusters 7, 9, and 18, (circle). **C)** By E11.0 (stage 37), the female left duct had further diversified into six clusters, Clusters 0, 1, 2, 5, 6 and 11 (circle). The cells of the E11.0 female right duct had also diversified into essentially the same five populations as the E11.0 left duct (circle).

Another discrete population of mesenchymal cells, comprising Clusters 3 and 10, was also present in E6.5 tissues, a population that persisted throughout duct development in both sexes (Figs 2A, 2B and 2C, S5D). This unique population has not previously been reported. Clusters 3 and 10 expressed the same MDM markers for the undifferentiated mesenchyme noted above (e.g., *VIM, OSR1, POSTN, EMX2*), but were transcriptionally distinct (Fig 2A). Clusters 3 and 10 were both enriched for a diagnostic set of gene transcripts, including *EBF1* and *FOXC2* transcription factors and G protein signaling-related factors *RERGL, ADGRA1* and *IGF1* (the receptors for which are widely expressed throughout the ducts). To validate this novel population, whole mount *in situ* hybridisation was carried out on female urogenital systems during duct formation (E6.5, E8.5, E11.0) and in the male urogenital system during duct regression (E8.5). Tissues were then sectioned to examine expression in transverse sections. *ADGRA1* and *EBF1* expression showed that these cells were located at the base of the Müllerian duct, adjacent to the mesonephric kidney (S5D Fig). Pseudotime analysis further confirmed that Clusters 3 and 10 were very transcriptionally distinct from the E6.5 MDM Cluster 17 (undifferentiated mesenchyme), which could suggest a difference in origin (Fig 5A). These cell populations remained present throughout development (Figs 2 and S5D), and the functional significance of these cells to duct structure and function requires further analysis.

The single population of mesenchyme identified in the E6.5 mixed sex ducts (Cluster 17) had expanded by E8.5. At this time point, the two sexes were harvested separately for analysis. The mesenchyme became sexually dimorphic, with unique populations emerging in males versus females. In the E8.5 female ducts, several mesenchymal clusters were recognised, indicating that the transcriptomic profile of the MDM had diversified as the duct developed (Fig 2B). The mesenchyme now made up 77.6% of the total cells in the ducts (vs 41.5% at E6.5). It comprised six cell populations, represented by Clusters 0, 1, 2, 5, 6 and 11 (Fig 2B). Most of the cells belonged to Clusters 1, 6 and 11, with a small proportion in Clusters, 0, 2 and 5. All of these sub-populations expressed the same mesenchymal markers found in the early “undifferentiated” MDM at E6.5 (i.e., *WNT4*, *EMX2, POSTN, OSR1, VIM* and *DMRT1)* (S7A and S7B Fig). In addition, novel transcripts were identified specifically in MDM Cluster 1, including the transcription factors *DMRT2*, *FOXE1*, *OSR2* and the growth factor, *HGF* (S7A Fig). These transcripts were not enriched in male MDM at the same stage. *FOXE1* and *HGF* were validated by *in situ* hybridisation and were strongly expressed in the female duct mesenchyme at E8.5 (Fig 3A). Furthermore, Cluster 1 highly expressed epithelial-to-mesenchymal transition genes such as *HMGA2, ZEB1, ZEB2, TWIST1 and TWIST2,* as at the previous developmental timepoint. Cluster 1 highly expressed genes promoting cell survival and proliferation, such as *SMARCA2*, which encodes a chromatin remodelling complex protein, and *MDK,* which encodes a secreted growth factor.

**Fig 3:**
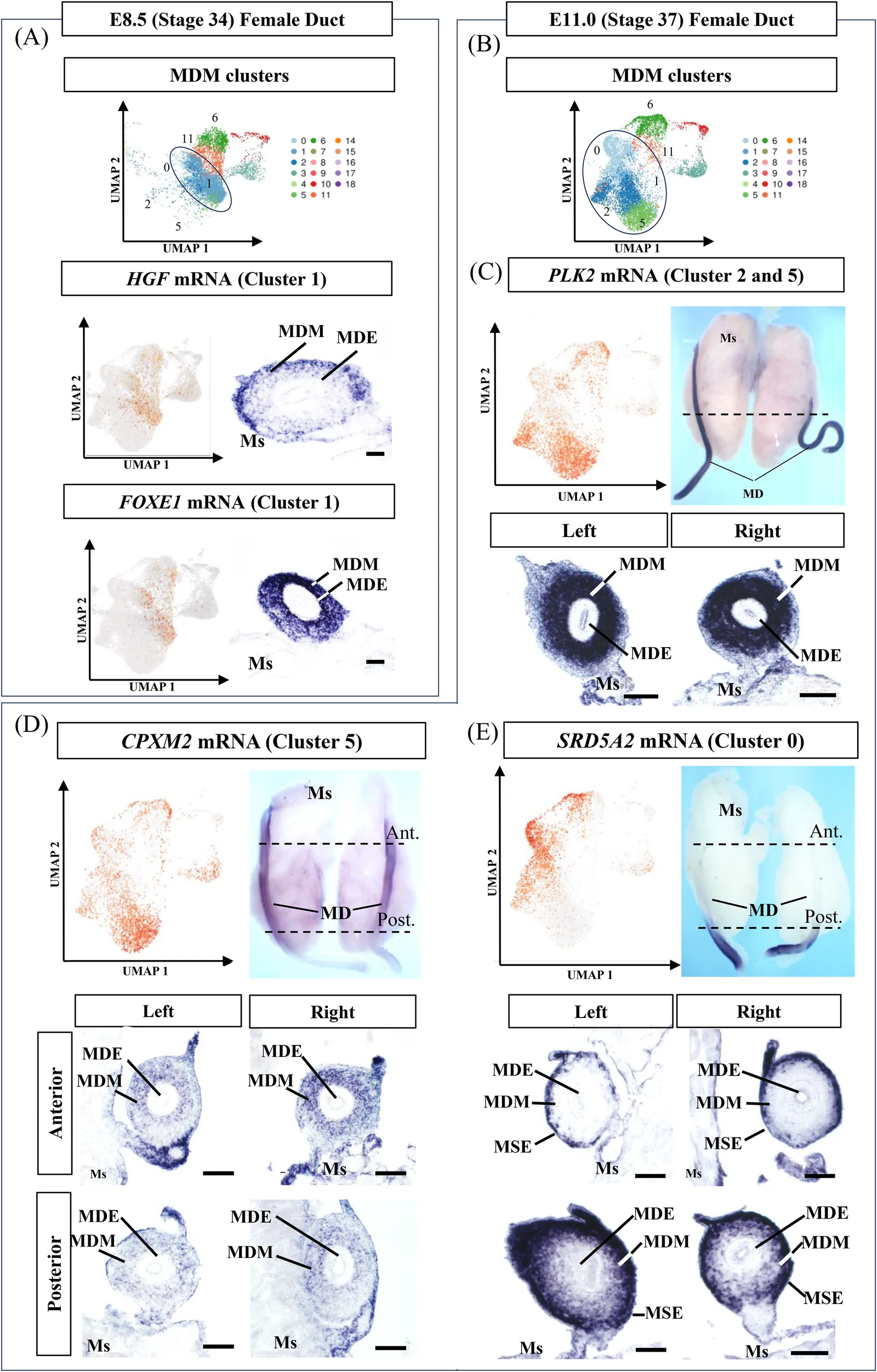
Mesenchymal populations in the E8.5 (stage 34) and E11.0 (stage 37) female duct. **(A)** The E8.5 female duct is enriched for MDM cluster 1 (circle). *HGF* and *FOXE1* are enriched in duct mesenchyme cluster 1 and are expressed throughout the mesenchyme (MDM) of the duct. **(B)** The E11.0 female left duct is enriched for MDM clusters 0, 2 and 5 (circle). **(C)** *PLK2,* enriched in MDM cluster 2 and 5, is expressed in a subset of MDM adjacent to the duct epithelium (MDE). **(D)** *CPXM2, e*nriched in MDM cluster 5, is expressed along the length of the duct excluding the caudal region. *CPXM2* is expressed in a subset of MDM adjacent to the MDE. **(E)** *SRD5A2, e*nriched in MDM cluster 0, is expressed in a subset of MDM adjacent to the coelomic epithelium (CE)., and is more strongly expressed in the caudal region of the duct. MD = Müllerian ducts. Ms = Mesonephric kidney. Scale bar = 50µm.

Cluster 6 was identified as a highly proliferative mesenchymal cell population, highly expressing genes associated with cell cycle progression and cell proliferation, such as *TOP2A*, *ASPM*, *FOXM1*, *MYBL2*, *CENPF,* (S7B Fig), consistent with the earlier cell phase analysis (S3B Fig, Female MDM). Cluster 11, located between Cluster 1 and 6 on the global UMAP, had lower levels of expression of transcripts that were enriched in either cluster 1 or 6 (e.g., *TOP2A* and *ELN*, S7C Fig). Cluster 11 was likely a transitional population of mesenchymal cells between quiescent and proliferative states (S7C and S3 Figs, Female MDM). At E8.5, Clusters 0, 2 and 5 had few cells, but these were greatly expanded at E11.0 (see below). Overall, the female mesenchyme at E8.5 was characterised by maintenance of a mesenchymal state, and great expansion of cell number and enhanced cell proliferation.

The female MDM showed further diversification at E11.0, corresponding to differentiation of the left duct and regression of the right duct (**S1****A and** **S1B Fig**). In the E11.0 female left duct, the same six clusters were recognised, clusters 0, 1, 2, 5, 6, and 11. However, Cluster 11 and 1 were greatly diminished in size, while Clusters 0, 2 and 5 had greatly expanded (Fig 2C). These latter clusters are considered to have derived from the E8.5 Cluster 1. Cluster 6 persisted as a proliferative population of mesenchyme. Differential gene expression analysis and *in situ* hybridisation was carried out on representative genes from these clusters, revealing that cell populations had diversified along the anterior-posterior axis of the duct. *PLK2* (a serine-threonine kinase) marked Clusters 2 and 5. It was strongly expressed along the length of both left and right ducts, and expression was restricted to an inner zone of MDM surrounding the MDE (Fig 3C). *CPXM2* (a carboxypeptidase) marked Cluster 5, and it was expressed along both ducts but excluded at the posterior poles (Fig 3D). Cluster 0 was marked by expression of *SRD5A2,* which encodes the androgen-producing 5-alpha reductase enzyme (Fig 3E). *SRD5A2* was strongly expressed throughout the MDM primarily in the posterior region of the left and right ducts (Fig 3E). In the anterior sections, *SRD5A2* expression was spatially restricted to an outer zone of mesenchyme adjacent to the MSE. (Fig 3E). Overall, spatial validation found that cell populations had diversified along the anterior-posterior axis, as well as the radial axis of the duct at E11.0. Notably, this molecular diversification of the duct precedes any morphological differentiation. Additionally, even though the right female duct undergoes regression at this timepoint (**S1A Fig**), the left and right duct showed no difference in staining intensity or expression pattern of the abovementioned markers.

In the chicken embryo, the right female Müllerian duct undergoes regression after E9 of development. By E11.0, the end point of analysis in the present study, right duct regression is advanced (S1A Fig). However, interestingly, the single cell RNA-seq data did not reveal any major transcriptional differences between the left differentiating and right regressing ducts. This is in sharp contrast to bilateral duct regression in the male at E8.5, where a unique population of regressing mesenchyme was identified (described below). Although considered to be mediated by AMH in both sexes, duct regression differs between the sexes. In males, both ducts disintegrate in a caudo-cranial direction, while in females the right duct “retracts” along the cranio-caudal axis (S1A Fig). Intriguingly, a unique regressing mesenchymal population or specific transcripts could not be identified in the right female regressing duct (S8A and S8B Fig). However, there was a lower proliferative index in the right regressing duct compared to the left duct (S8D Fig). Additionally, this study performed scRNA-seq analysis of isolated anterior tips of right female ducts, and we did detect reduced expression of some anterior *HOX* gene cluster members, such as *HOXC10,* at the anterior tips of the regressing right female ducts (S8C Fig). Therefore, regression of the right duct in females could involve disrupted *HOX* gene expression, a very different pattern of regression compared to that observed in males.

### Transcriptional characterisation of bilateral duct regression in males

In male chicken embryos, both left and right Müllerian ducts undergo regression, mediated by AMH, that is morphologically apparent from E8.5 (See S1 Fig). We found that the E8.5 male duct mesenchyme is transcriptionally very divergent from that in females, and that duct regression involves two distinct mesenchymal cell populations. Furthermore, we characterise the molecular signatures of these populations. As in females, the male Müllerian duct mesenchyme was identified through the expression of known mesenchyme markers, such as *DMRT1, WNT4, OSR1, COL1A2, HTRA3,* and the absence of *LHX1, WNT7A or CDH* (E-cadherin) [28,30,36,40,46,64,65]. At E8.5 the male mesenchyme acquired a unique, sexually dimorphic molecular signature, expressing the novel transcription factors *MSX1, MSX2, RUNX2, GATA5, DLX*3 and *DLX5,* and the signaling regulators *DKK4* and *NRG1* (Neuregulin) (Figs 4A and S10C). None of these genes were significantly expressed in female mesenchyme. The male regressing mesenchymal cells comprised three distinct clusters (Clusters 7, 9 and 18), and they were all absent in female Müllerian ducts (Fig 4). One cluster represented a small group of proliferative cells (S3 Fig). The other two Clusters (7 and 9) represented the regressing male mesenchyme, based on known markers of Müllerian duct regression, such as *AMHR2*, *RUNX2, DLX5/6* and the AMH downstream effectors, *SMAD6,* matrix metalloprotease, *ADAMTS19* and metalloprotease-disintegrin *ADAM12* [28,66]. Differential gene expression analysis showed that these two regressing mesenchymal populations were transcriptionally distinct (Figs 4A and S10C). Both clusters expressed the *MSX2* transcription factor. Cluster 9 also highly expressed *MSX1/RUNX2/GATA5/DKK4/SMAD6/DLX3/DLX5* (Figs 4A and S10C). Cluster 7 expressed lower levels of the above genes and instead was enriched for expression of *NRG1* (Neuregulin), *RSPO3* (A WNT agonist), *OSGIN1* (an Oxidative stress activated cell death inducer)*, LVRN* (Laeverin, encodes an enzyme involved in catabolism) and *HGF* (Hepatocyte Growth Factor) (Figs 4A and S10C). The above genes are novel candidate regulators of Müllerian duct regression. Notably, Clusters 7 and 9 were clearly defined, respectively, as an *MSX2 + /MSX1-* population and *MSX2 + /MSX1 +* population (Fig 4A). *MSX1/2* are highly conserved homeobox transcription factors. Another transcript limited to these populations was *DKK4*. *DKK4* encodes an inhibitor of the canonical WNT/β‐catenin pathway, and was expressed in all cells of Cluster 9, and a subset of Cluster 7 cells (Fig 4A).

**Fig 4:**
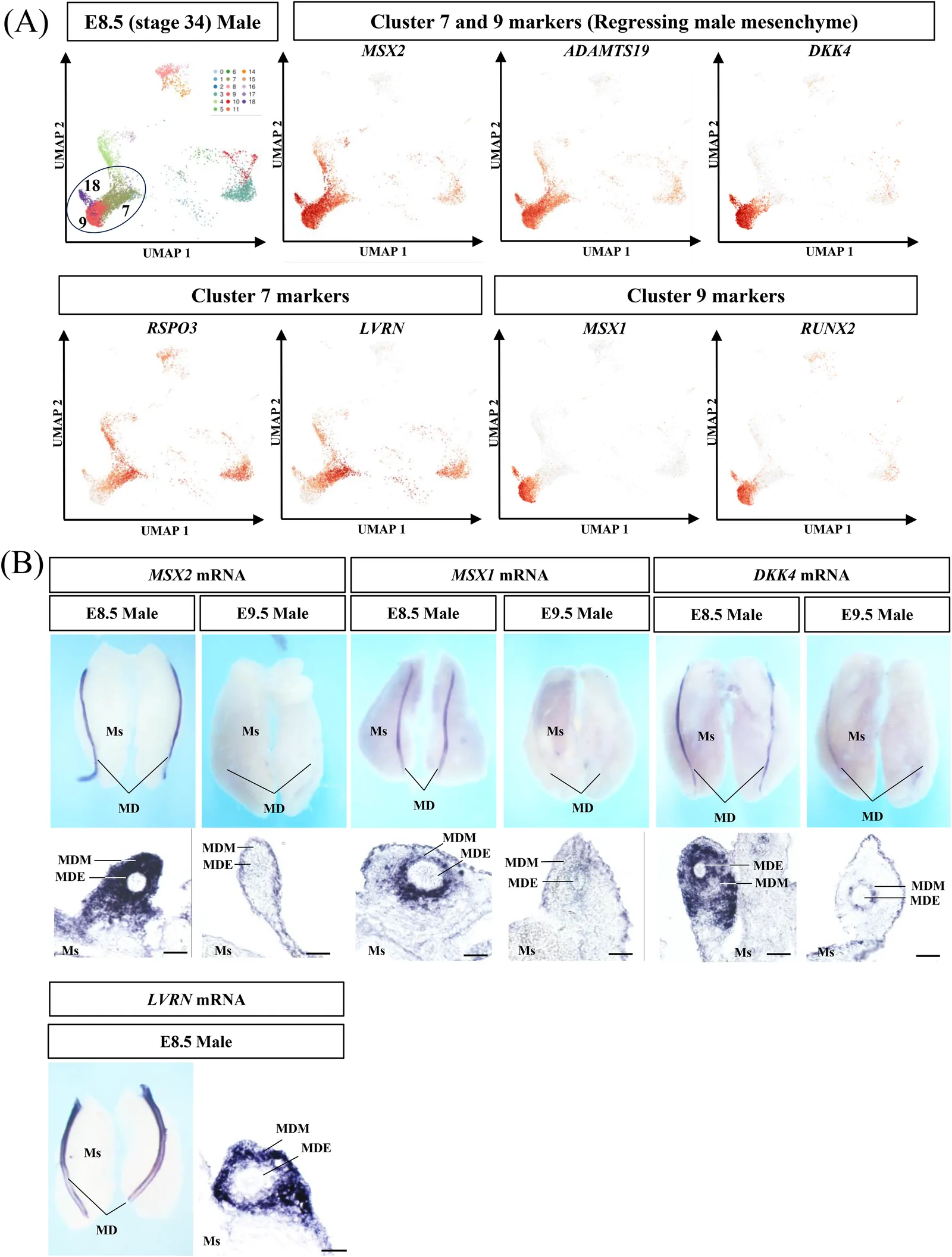
Mesenchymal populations in the E8.5 (stage 34) male duct. **A)** UMAPS of transcripts expressed in mesenchymal populations of the regressing male duct (**B)** *MSX2,* is expressed throughout the mesenchyme (MDM) of the regressing duct at E8.5. *MSX2* expression is absent once duct regression is complete at E9.5. *MSX1* is expressed in a subset of MDM at E8.5, in an inner ring of MDM located adjacent to the duct epithelium (MDE). *MSX1* expression is weaker at E9.5. *DKK4* is expressed in a mosaic pattern in a subset of the regressing duct mesenchyme at E8.5. *DKK4* is weakly expressed in the MDM flanking the MDE at E9.5. *LVRN* expression is lower in the cranial region of the male duct at E8.5. *LVRN* expression is lower in the inner ring of mesenchyme adjacent to the MDE. MD = Müllerian ducts. Ms = Mesonephric kidney. Scale bar = 50µm.

To validate gene expression the male MDM transcriptional clusters identified by single cell RNA sequencing, whole-mount *in situ* hybridisation was used. This was carried out on male urogenital systems during (E8.5) and after (E9.5) Müllerian duct regression (Fig 4B). This expression analysis showed that *MSX1*, *MSX2* and *DKK4* were spatially restricted to a subset of male Müllerian duct mesenchyme. (Fig 4B). *MSX2* was strongly expressed throughout the mesenchyme of the regressing male duct at E8.5 and was downregulated of the degenerating duct at E9.5 (Fig 4B). *MSX1* was strongly expressed in a subset of MDM cells and became spatially restricted to a ring of mesenchyme surrounding the inner MDE. *MSX1* was more weakly expressed in the regressed male duct at E9.5 (Fig 4B). *DKK4* was strongly expressed in a subset of the MDM, including the ring of mesenchyme immediately around the MDE, as well as in non-spatially restricted patches throughout the MDM. In the E9.5 male duct, in which regression was advanced, *DKK4* was weakly expressed in the MDM adjacent to the MDE (Fig 4B). The other population of male-specific mesenchyme was marked by the expression of *LVRN* (Laeverin). This population localised to an outer ring of MDM adjacent to the MSE (Fig 4B). Hence, the two male MDM populations were spatially distinct, comprising an *MSX2 + /MSX1 + //LRVN-* inner population and a *MSX2 + /MSX1-/LRVN+* outer population. *MSX2* and *MSX1* were not expressed in the female MDM, except in the most posterior MDE (S6C Fig).

Pseudotime analysis was carried out to elucidate the lineage trajectories of the E6.5 mesenchyme. A UMAP was generated, consisting only of the MDM cell populations in all samples (Fig 5A). Using Palantir, the mesenchymal population at E6.5, Cluster 17, was set as the starting point for lineage tracing (Fig 5A). Pseudotime analysis traced the mesenchymal cells to two terminal states, and calculated two corresponding developmental trajectories, designated C1 and C2 (Fig 5A). With reference to the UMAP, C1 was determined to be the differentiating mesenchyme of the female lineage. C2 corresponded to the regressing mesenchyme in the E8.5 male lineage (Fig 5A). The pseudotime analysis based on transcript profiling reconstructed the developmental trajectory of the duct mesenchyme and illustrated a clear developmental split in the male and female ducts after E6.5. In the female lineage trajectory, the E6.5 mesenchyme gave rise to Cluster 1 of the E8.5 female mesenchyme, which in turn gave rise to Clusters 0, 2 and 5 of the E11.0 female mesenchyme. In the male lineage trajectory, the E6.5 mesenchyme gave rise to the E8.5 male mesenchyme Clusters 7, 9 and 18. The male-specific *MSX2 + /MSX1-/LRVN+* outer MDM population (Cluster 7) represented a transitional state, giving rise to the *MSX2 + /MSX1 + /LRVN-* inner population (Cluster 9) (Fig 5A). As the inner population expressed AMHR2, we consider this to be the population undergoing regression, with cells of the “outer” mesenchyme being recruited to the inner regressing fate.

**Fig 5:**
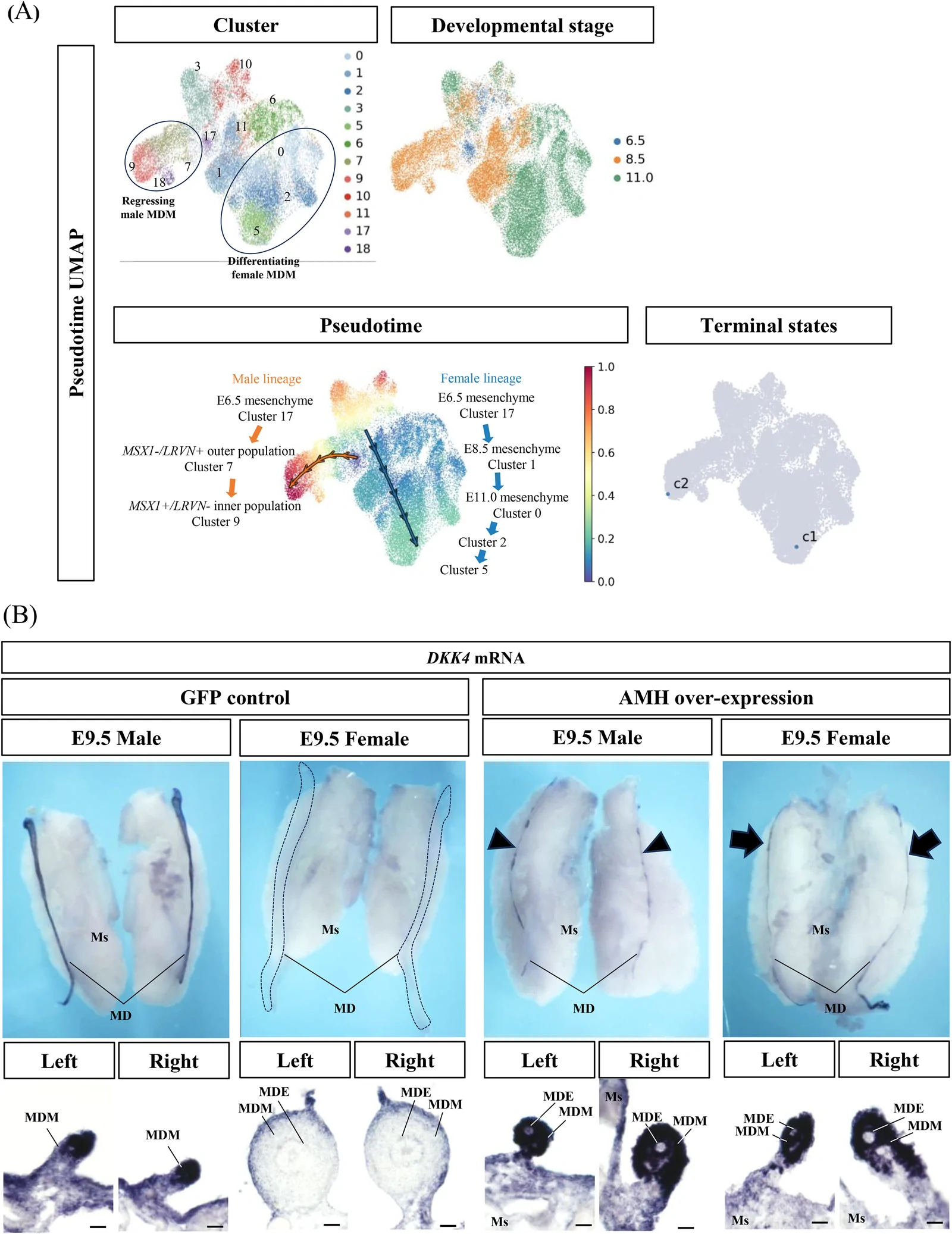
**(A) Cell lineage trajectory analysis of Müllerian duct mesenchyme cells (MDM).** Cells are color-coded by cluster or developmental stage (top row). The arrows delineate inferred developmental trend over pseudotime, in which the E6.5 mesenchyme undergoes a developmental split into male and female lineages. The male lineage is characterized by an intermediate MSX1-/LVRN+ population (Cluster 7), and transitions into a MSX1 + /LVRN- population (Cluster 9). **(B)** Effect of viral-mediated AMH over-expression on duct morphology and *DKK4* expression**.** In controls expressing GFP, *DKK4* is expressed in the mesenchyme (MDM) of male but not female ducts at E9.5. Meanwhile, AMH over-expression induced duct regression and ectopic *DKK4* expression in the MDM of female ducts (arrows). In males, ectopic AMH accelerates duct regression (arrowheads). MD = Müllerian ducts. Ms = Mesonephric kidney. Scale bar = 50µm.

### Functional analysis of *DKK4* reveals a role in Müllerian duct regression

The present study identified the WNT signaling regulator *DKK4* as a novel gene transcript enriched in the mesenchyme of regressing male Müllerian ducts at E8.5. To assess the potential role of this gene in chicken Müllerian duct regression, functional studies were conducted *in ovo*. Firstly, AMH was mis-expressed in female embryos, using the RCASBP viral vector, delivered to embryos at the blastoderm stage. AMH is normally lowly expressed in female gonads, versus highly expressed in male gonads, commensurate with duct regression. Successful mis-expression was confirmed by AMH *in situ* hybridisation at E9.5, which showed robust *AMH* mRNA expression in the gonads and mesonephric kidneys of male and female embryos, compared to controls infected with virus expressing GFP (S11 Fig). As reported previously [67], mis-expression of *AMH* in female chicken embryos results in masculinisation of the gonads, such that they appear testicular (S11 Fig). Mis-expression of AMH in female embryos resulted in duct regression and ectopic activation of *DKK4* transcription (Fig 5B). This indicates that *DKK4* expression responds to AMH hormonal signaling in the Müllerian duct. We then assessed the impact of *DKK4* mis-expression on duct development in both sexes. Electroporation was used, expressing *CAGS→GFP-T2A-DKK4* from a *TOL2* based integrating plasmid. A *TOL2(CAGS→GFP)* construct was used as a control. These plasmids were electroporated into the left intermediate mesoderm of E2.5 embryos in the region of the presumptive Müllerian duct and tissues were examined at E9.5. Successful unilateral electroporation was confirmed using the GFP reporter. Robust GFP expression was observed in the left duct of both GFP control and *DKK4* over-expressing tissues (Fig 6A and 6B).

**Fig 6:**
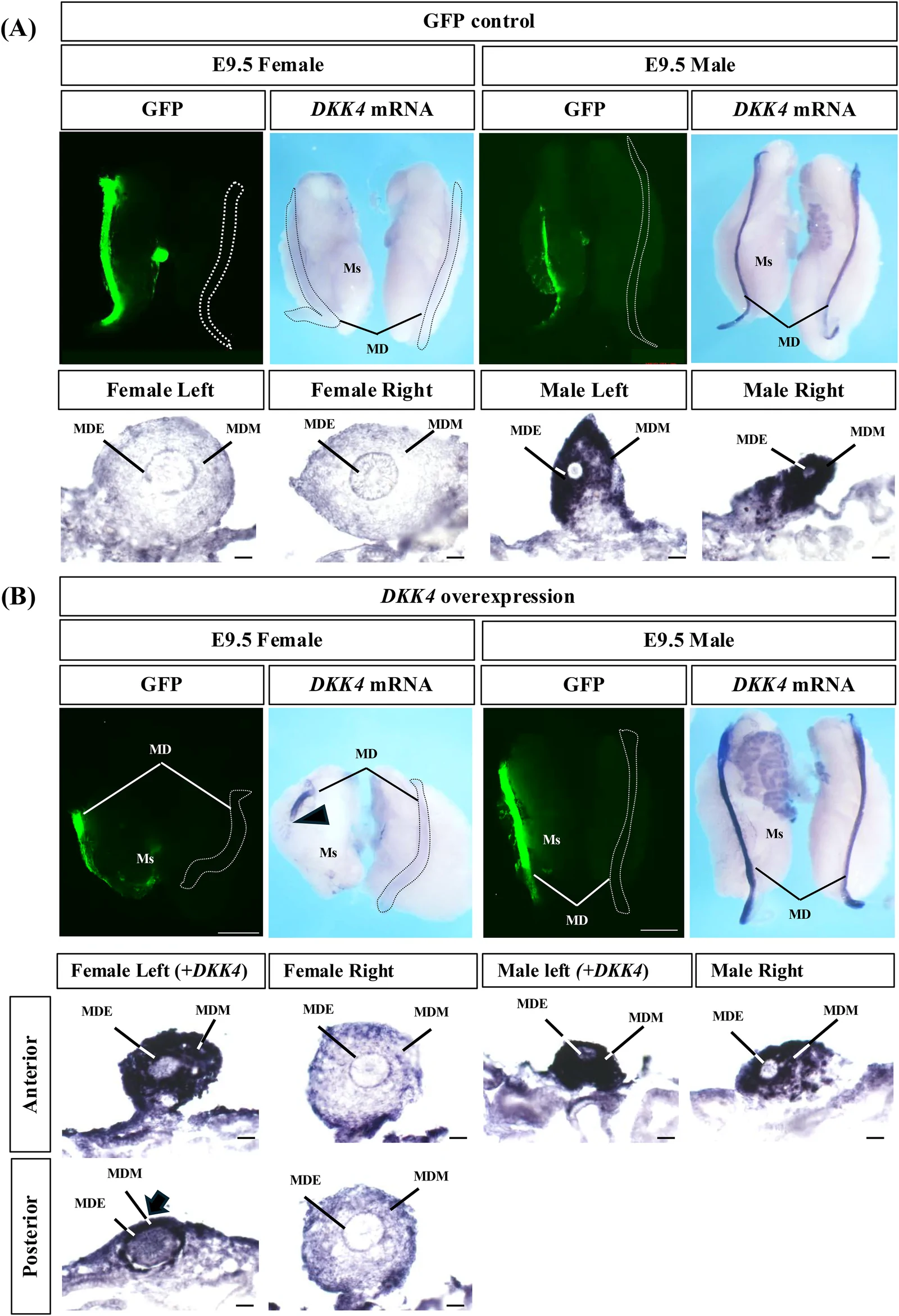
Effect of *DKK4* overexpression on Müllerian duct development. **(A)** Duct morphology and DKK4 expression in control urogenital systems expressing GFP. In E9.5 females, both left and right ducts were robust and lacked DKK4 expression. In E9.5 males, both ducts were regressed and DKK4 was strongly expressed in the mesenchyme (MDM). **(B)** Duct morphology and DKK4 expression following unilateral DKK4 overexpression in the left Müllerian duct. In the E9.5 female, ectopic *DKK4* expression was induced in the left duct, which caused cranio-caudal regression of the duct (arrowhead). Transverse sections showed a reduced mesenchymal field in the posterior region of the left duct (arrow). In E9.5 males, both ducts were regressed, and DKK4 was strongly expressed in the MDM. No morphological differences were noted compared to the GFP controls. White scale bar = 1000µm. Black scale bar = 25µm.

Duct development was assessed by whole mount *in situ* hybridisation for *DKK4* as well as the mesenchyme marker *WNT4.* In female tissues electroporated with GFP, both the left and right ducts were morphologically well-developed, and no *DKK4* expression was observed (Fig 6A). Robust *WNT4* expression was observed in the mesenchyme of both ducts (S12 Fig). In females mis-expressing *DKK4*, the gene was strongly expressed in the electroporated left duct, and remained absent from the un-electroporated right duct, as expected (Fig 6A and 6B). Ectopic *DKK4* mis-expression induced significant regression of the left duct in female embryos (Fig 6B and 6C). Furthermore, the regression appeared to proceed in the caudo-cranial direction, which is characteristic of male duct regression. In 10 out of 17 *DKK4*-electroporated females, the anterior pole of the left duct remained well-developed, while the posterior region was significantly regressed (S13 Fig). In the remaining individuals, the left electroporated duct was regressed along its entire length (Figs 6B, 6C and S13). Furthermore, staining for the mesenchymal marker *WNT4* showed a loss of mesenchyme in the electroporated duct (Fig 7A and 7B). Total duct area, MDM area, MDE area, and WNT4 staining intensity were quantified using FIJI image analysis of *in situ*-stained tissue sections. The cross-sectional area of the entire duct was significantly reduced (p = 0.0184), as was the duct epithelium (p = 0.0323) and mesenchyme (p = 0.0298). WNT4 expression in the mesenchyme, measured by mean pixel intensity, was unchanged (p = 0.5252) following *DKK4* over-expression. This revealed that the ducts and their compartments were significantly smaller after DKK4 over-expression compared to controls. In male tissues, both *GFP* and *DKK4* electroporation had no impact on duct development. Both the left and right ducts had thin morphology, as expected of ducts undergoing bilateral duct regression (Fig 7A). Additionally, the *DKK4* overexpression and control tissues strongly expressed *DKK4* in both left and right male ducts (Fig 6A and 6B). Both ducts had thin morphology, and weak expression of *WNT4* in the duct mesenchyme, consistent with regression (S12 Fig). Lastly, DKK4 over-expression at E2.5 did not block early duct development. Tissues examined at E5.5, 3 days after DKK4 delivery at E2.5, showed normal duct formation (S14 Fig), which indicates that DKK4 over-expression has a later, specific effect upon duct regression.

**Fig 7:**
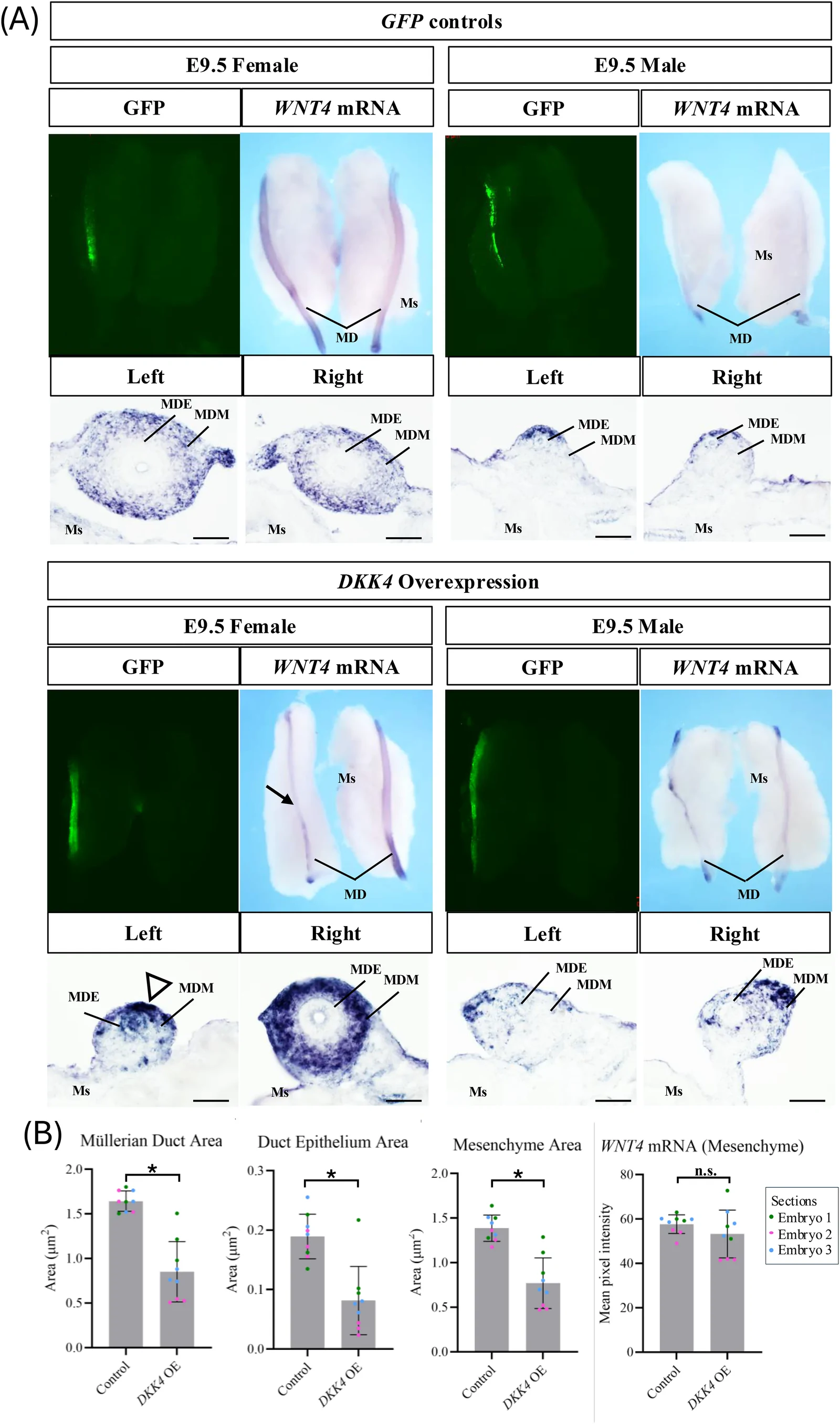
WNT4 mRNA expression and duct morphology at E9.5 (stage 35) following DKK4 over-expression in the left Müllerian duct. **(A)** In the GFP controls, both left and right female ducts are thick and express WNT4 throughout the duct mesenchyme (MDM). In the male, the regressing ducts are thin and WNT4 is weakly expressed in the MDM. Following DKK4 overexpression of the left female duct, WNT4 is expressed in the MDM, and the duct is thin (arrow) due to a loss of mesenchyme (arrowhead). In the male left duct, WNT4 expression and duct morphology is unchanged following DKK4 overexpression. MDE = Müllerian duct epithelium. Ms = Mesonephros. Scale bar = 50µm. **(B)** Transverse section area of the electroporated left duct in E9.5 females. Bar graphs indicate mean area and standard deviation while scatter plot represents transverse sections, colour coded by embryo (n = 3 each for GFP control and DKK4 overexpression). Following DKK4 overexpression, transverse area of the duct is significantly reduced (p = 0.0184), as is the duct epithelium (p = 0.0323) and mesenchyme area (p = 0.0298). WNT4 expression in the mesenchyme, measured by mean pixel intensity, is unchanged (p = 0.5252) following DKK4 overexpression.

## Discussion

This study sheds new light on the biology of Müllerian duct sexual differentiation. Firstly, we show that the Müllerian ducts of the chicken embryo are not sexually dimorphic at the transcriptional level up to the time of overt morphological differentiation at E8.5. We find that there is greater cellular complexity in the differentiating ducts than previously considered, and we define molecular signatures of Müllerian duct sexual differentiation (Fig 8). We expand the molecular markers of the MSE (Müllerian Surface Epithelium), the MDE (Müllerian Duct Epithelium) and the MDM (Müllerian duct Mesenchyme). We define the molecular signature and lineage trajectory of Müllerian duct regression in males and show that this regression is regulated in a fundamentally different way to unilateral duct regression in females. Lastly, we functionally analyse a novel gene in Müllerian duct biology, *DKK4,* showing that it plays a role in duct regression.

**Fig 8:**
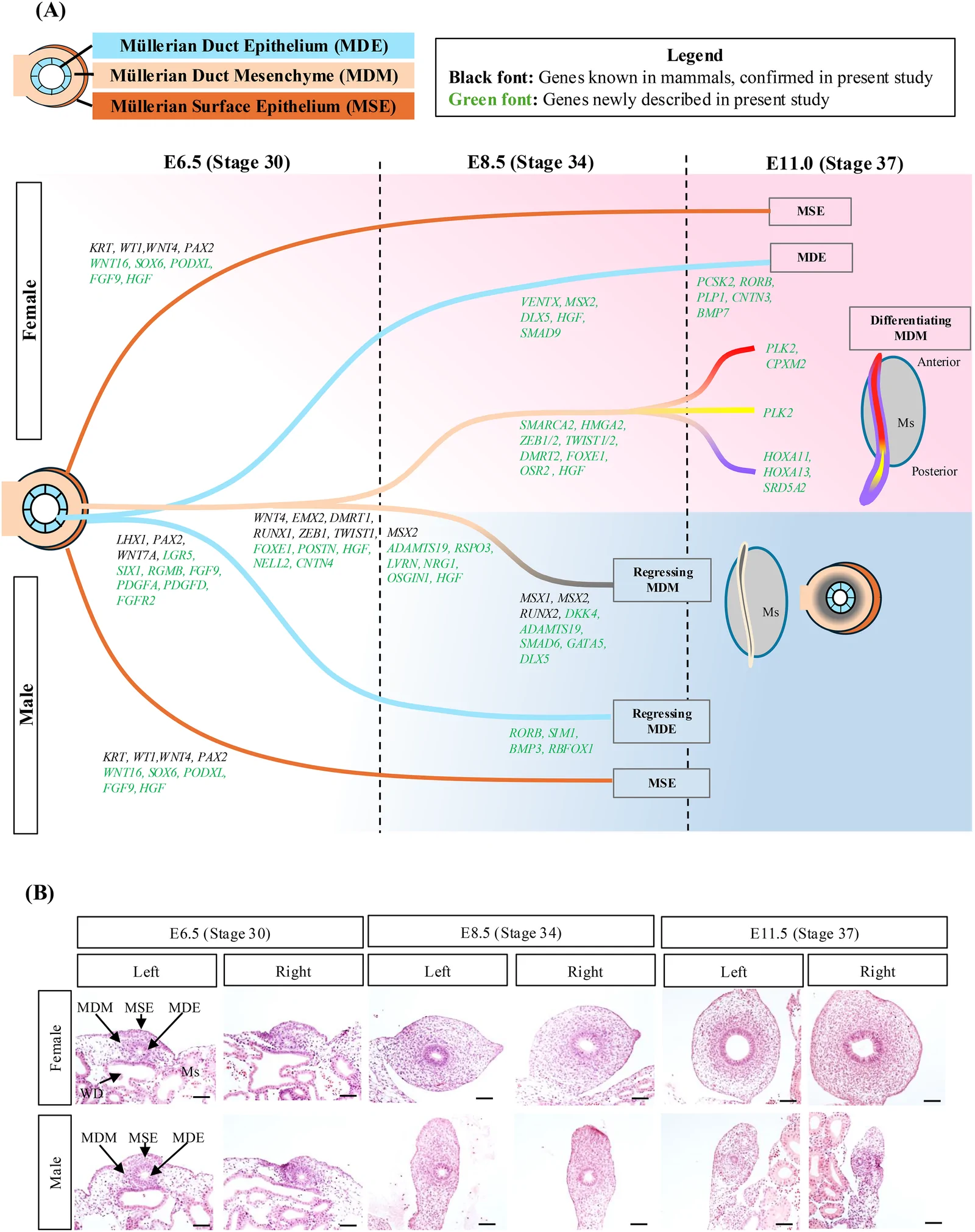
Schematic representation of cell lineage specification in the chicken Müllerian duct, based on single-cell transcriptomics and pseudotime lineage tracing. **(A)** The lines represent distinct cell populations, while the green font indicates novel molecular signatures identified in the present study. The duct mesenchyme (MDM) at E6.5 is sexually undifferentiated and expresses novel duct elongation genes such as *FOXE1.* At E8.5, the female MDM is enriched for transcripts promoting cell survival and proliferation such as *HGF.* In the regressing male, an *MSX1 + /MSX2 +* population is present the inner MDM adjacent to the MDE, while an *MSX1-/MSX2 + /LVRN+* population is present in the outer MDM. These populations represent a more advanced and less advanced stage of regression, respectively. At E11.0, the female MDM diversifies, leading to regionalisation along the anterior-posterior and radial axes of the duct. In the inner MDM, a *CPXM2 + /PLK2 +* population (red) is present in the anterior region of the duct, while a *CPXM2-/PLK2 +* population (yellow) is present in the posterior region. A SRD5A2 + population is present in the outer MDM, and *SRD5A2* expression is higher in the posterior region of the duct (purple). Created in BioRender. Major, **A.** (2026) https://BioRender.com/36i9apw. **(B)** Corresponding histology of the Müllerian ducts during sexual differentiation.

The MSE, the outer surface epithelium of the developing duct, is defined by *KRTs/ WT1/ PAX2/ WNT16/ SOX6/ PODXL/ WNT4/ FGF9* and *HGF* in both sexes at E6.5 (prior to duct sexual differentiation) (Figs 8 and S4B, S4C and S4D). Lineage tracing and genetic studies have shown that cells of the MSE gives rise to the entire Müllerian duct, to both the inner duct epithelium (MDE) and the duct mesenchyme (MDM), the former through a partial EMT and the later a full EMT [30,39,40,46]. Previous studies based on mouse and chicken have suggested that this Müllerian surface epithelium, the MSE, has two sub-populations. First, a group of meso-epithelial precursor cells expressing *PAX2/8/EMX2/LHX1/WNT7A* that gives rise to the MDE, and another, *WNT4/DMRT+* population that gives rise to the MDM [28]. We did not detect such distinct populations in the current study, although this may be due to the stage examined. The earliest stage studied here was E6.5, when the MDE and MDM were already present. The two putative subpopulations may have formed and expressed those markers prior to this time, though, if so, the markers were not maintained in distinct populations by E6.5. Similarly, *LHX1* has been shown to be required in the MSE of mouse and chicken embryos at early stages, before HH stage 30 and prior to duct invagination [36,42]. In our study, *LHX1* mRNA was detected only in the MDE, not the MSE, which could be a difference in timing.

Sexually dimorphic gene expression was observed at E8.5, at which time both the MDE and the MDM exhibited sexually divergent molecular fates (Fig 8). Several previous studies in chicken and mouse have shown that MDE, a meso-epithelial tube, derives from the MSE via invagination and cell proliferation, followed by migration in a craniocaudal direction [39,46]. Here, we identified three sub-populations of related MDE cells at E6.5. We define the molecular signature with the following signaling transcripts enriched in the E6.5 MDE: *LGR5* (a stem cell marker), *PDGFA* and *PDGFD* (Plate-Derived Growth Factors), *FGFR2, BMPR1B*, the transcription factor *SIX1*, and the epithelial adhesion factor, *EPCAM* (Fig 7). At E8.5, the MDE showed marked signs of sexually dimorphic gene expression (S6A Fig) consistent with the onset of morphological sexual differentiation, where the male ducts start to regress. In males, the MDE exhibited a molecular signature characterised by expression of the transcription factors *SIM1* and *RORB* (Retinoic acid signaling-related nuclear receptor), and signaling molecules *BMP3*, *BMPER* and the RNA-binding splicing factor, *RBFOX1* (Figs 8A and S6A). This coincides with the onset of morphological duct regression. These genes are candidate regulators or mediators of male MDE regression, as they were not enriched in females. Müllerian duct regression is directed by AMH, secreted by the embryonic testis in mammals and by both the testis and ovary in birds, though at a lower level in females ^9,86,100^. Here, also we identified a molecular signature in female MDE but not male MDE at E8.5: *DLX5/MSX2/VENTX/HGF* and *SMAD9* (Figs 8A and S6A). These factors are novel candidate regulators of MDE development in females as opposed to regression in the avian model.

At E6.5, the MDM expressed known diagnostic duct mesenchyme markers such as the transcription factors *DMRT1, EMX2*, *RUNX1* [33,45,68*]*, together with novel markers such as *FOXE1* and *HGF* (Fig 3A), and EMT markers such as *ZEB1* and *TWIST1* (S5B Fig). By E8.5, clear sexual dimorphism had emerged in the MDM at the molecular level, as the male showed regression while the female showed further development. Our study enriches our understanding of Müllerian duct regression in male embryos, identifying more than one homogeneous mesenchymal cell population (MDM) and uncovering new candidate genes that regulate regression. In the chicken embryo, this male MDM regression begins at E8.0 (HH stages 33–34) [55,65,69]; the molecular data reported here supports this time frame. The study reported here shows that the regressing mesenchyme of the male chicken Müllerian duct has a molecular signature comprising *MSX1, MSX2, RUNX2, OSGIN1, GATA5, DLX*3 and *DLX5,* and the signaling regulators *DKK4* and *NRG1* (Neuregulin) (Fig 7). A previous subtractive hybridisation screen in the chicken embryo identified the muscle homeobox gene *MSX1/2* as candidate regulators of male duct regression, though the cellular location and functional studies were not reported [70]. The distal-less-related transcription factors Dlx5 and Dlx6 have also recently been implicated in duct mesenchyme regression in male mice [66], pointing to a conserved role in the MDM. Here, we identify additional novel duct regression regulators, including the stem cell factor *NRG1*, the transcription factors *GATA5*, and the WNT-signaling modulator, *DKK4*, which we functionally analysed. None of these factors were expressed at a significant level in female chicken MDM and are hence considered direct or indirect targets of AMH action during male duct regression. *NRG1* and *GATA5* require further functional analysis.

The process of male duct regression begins with AMHRII signaling in the MDM. Mouse studies have shown that regression requires expression of AMHRII in the mesenchyme and expression of *WNT7A* is the adjacent duct epithelium (MDE) to send inductive signals that stimulate AMHRII expression [5,13,71]. We found that the regressing male mesenchyme is not a homogenous population of cells. It comprises three transcriptionally distinct populations, two closely related and one that was a proliferating population (despite regression of the MDM as whole). Cells in the first two populations (Clusters 7 and 9) both expressed “regression markers” such as ADAMTS19, but they also expressed some unique or enriched genes. Cluster 9 strongly expressed *MSX1* and *DKK4*, which were not expressed or lowly expressed in the other mesenchymal population, Cluster 7 (Fig 4A and 4B). Conversely, Cluster 7 was enriched for a subset of genes such as *LVRN*, which, when analysed by *in situ* hybridisation, confirmed enrichment in the outer mesenchymal layer (Fig 4A and 4B). This outer layer also shared expression of *MSX2* with the inner mesenchymal layer. Spatial validation showed that the Cluster 9 enriched transcripts, *MSX1* and *DKK4*, were confined to mesenchyme surrounding the inner Müllerian duct epithelium (MDE) at E8.5 and E9.5 (Fig 4B). The cell population represented by Cluster 7, was a more peripheral mesenchymal population, it did not express *MSX1*, but expressed *RSPO3* (a WNT signaling activator) and *LVRN* [72] (Fig 4B). Pseudotime trajectory analysis of the MDM showed that the *MSX1-*/*LRVN + /*DKK4-/RUNX2- Cluster 7 was transitional between the undifferentiated E6.5 duct mesenchyme and the *MSX1 + /DKK4 + /RUNX2 +* Cluster 9 (Fig 5A). This indicates that the two populations represent two developmental states, one more advanced in regression (Cluster 9) compared to the other (Cluster 7) (Fig 7). This may reflect the closer proximity of the mesenchyme of Cluster 9 to the MDE, which secretes WNT7A to activate *AMHRII* in the MDM and so directs duct regression, at least in mouse [13,30,73]. It would be of interest to extend the single cell transcriptomic analysis of regressing male ducts beyond E8.5, to include the subsequent fate of these two mesenchymal populations and to document regression of the MDE.

In this study, we identified *DKK4* (*Dickkopf-related protein 4*) as a new factor implicated in male Müllerian duct regression. *DKK4* expression was male-specific, most strongly expressed in the inner MDM, immediately adjacent to the MDE (Fig 4B). In the present study, *DKK4* expression was ectopically induced in female embryos over-expressing AMH (Fig 5B), indicating that *DKK4* can respond to AMH, either directly or indirectly. Furthermore, unilateral ectopic expression of *DKK4* induces striking left duct regression in females and loss of duct mesenchyme, as assessed by *WNT4* mRNA expression (Figs 6 and 7). *DKK4* encodes a member of the dickkopf family of cysteine-rich cell surface glycoproteins that act as potent inhibitors of canonical WNT/β-catenin signaling [74]. This inhibition is mediated via DKK protein binding to the obligate WNT co-receptor, LRP5/6 [75–78]. DKK family members can modulate a variety of developmental processes regulated by WNTs, including cellular differentiation, cell fate determination, organogenesis, mitogenesis and stem cell maintenance [79–82]. It is known that WNT signaling is required for proper Müllerian duct formation and sexual differentiation in response to AMH (reviewed in [64]. In the mouse, *WNT4* is expressed in the early duct mesenchyme, and targeted deletion of either *WNT4* or its intracellular transducer, the beta-catenin gene (*Ctnnb1*), results in the failure of proper duct formation [46,47,83]. In the chicken embryo, expression analysis points to a comparable essential role for WNT4 in duct formation. During the sexual differentiation phase of Müllerian duct development in mouse, WNT signaling is important in both sexes. In males, MDE-derived WNT7a triggers *AmhrII* expression in the MD, leading to duct regression while, in females *WNT7a* is required for further duct differentiation [71,84,85]. Here, *DKK4* may play a modulatory role in precisely regulating that level of WNT signaling required for Müllerian duct regression in males. In mouse, for example, both inactivation as well as constitutive activation of *Ctnnb1* (β-catenin) in the mesenchyme leads to duct retention, suggesting that tight regulation of *Ctnnb1* is crucial for duct regression [53,86,87]. In the chicken, DKK4 may have a modulatory role on β-catenin activity. Further, it may be specifically influencing WNT7A, as opposed to WNT4 earlier in duct development. In mice, WNT7a prevents cell death of the female reproductive tract [84], and so ectopically expressing the WNT inhibitor, DKK4, may allow apoptosis to proceed in the female duct, as occurs in males. Another WNT inhibitor, Wif1 (WNT-Inhibitory Factor 1) has also been shown to play a role in Müllerian duct, being male-specific and not expressed in *AmhrII* null mice [88]. Knockdown of *Wif1 in vitro* inhibits proper duct regression, implying that Wif1 is needed to finetune the amount of WNT signaling required for duct regression. It would be of interest here to assess levels of stabilised cytoplasmic β-catenin after DKK4 mis-expression, and to examine the effects of *DKK4* knockdown or loss on duct regression in chicken. Lastly, DKK family members can have non-canonical functions, independent of WNT signaling, such via activation of PI3K/AKT signaling and activation of intrinsic pro-apoptosis pathways [89,90]. This could apply here to DKK4 in the Mullerian duct.

In the female chicken embryo, the MDM follows a different developmental fate compared to that observed in males. In the left and right ducts, both the MDM and MDE expand through cell proliferation (S3 and S1B Fig). Here, at E8.5, the female MDM develops a molecular signature comprising *SMARCA2/HMGA2/ZEB1/2/TWIST1/2/DMRT2* and persistent expression of *FOXE1, OSR2* and *HGF* (Hepatocyte Growth Factor) (Fig 8). These genes are candidate regulators of the female MDM. Subsequently, the left duct continues to develop, while the right duct begins to regress. To compare the transcriptional changes between the left ducts, single cell RNA-seq was performed on left vs right ducts, as well as the anterior tips of the right duct. At E11.0, both left and right female ducts comprised several clusters of transcriptionally distinct cells (Fig 3B). Validation of markers expressed in these clusters by *in situ* hybridisation showed that these clusters reflected different cellular populations along the length of the Müllerian duct (Fig 3C, 3D and 3E). This must reflect spatial pre-patterning of the duct prior to later regionalisation. In the chicken, the Müllerian duct develops into the oviduct, an organ that becomes functionally segmented into infundibulum, magnum, isthmus, uterus (shell gland) and vagina [41]. Homologous regions form in the mouse oviduct. This study observed restricted expression of *HOX* gene clusters, as well as the candidate genes *CPXM2* and *SRD5A2* in the single-cell data (S9 Fig). This is consistent with previous studies showing that posterior *HOXA* and *HOXD* genes are involved in regionalised differentiation of the mammalian Müllerian duct [15,41,91–96]. In mouse, the oviduct is regionally patterned postnatally via spatially regulated *Hox* gene expression, while we found regionalised *Hox* expression in the chicken already at the embryonic stages (S9 Fig). Differential gene expression along the length of the female Müllerian duct indicates that there is molecular differentiation preceding overt morphological differentiation.

In contrast to AMH-mediated bilateral Müllerian duct regression in male chicken embryos, females show an unusual pattern of left duct differentiation and right duct regression (S1A Fig). This phenomenon in chicken is unique to birds and can inform our understanding of Müllerian duct biology. Right duct regression females is thought to be mediated by gonadal-derived AMH, because the embryonic female chicken gonad produces AMH, albeit lowly compared to male [57–59] and testis grafts or exogenous AMH can induce bilateral duct regression in female chicken embryos (Fig 5B) [97]. This shows that both left and right female ducts can respond to AMH. However, the pattern of normal right duct regression in female chicken embryos differs from that seen in males. In females, unilateral right duct regression progresses in a craniocaudal fashion, from anterior to posterior (S1 Fig). In males, the converse applies; both ducts regress in a caudo-cranial manner, from posterior to anterior (S1 Fig) [34]. Intriguingly, we did not detect a specific molecular signature of right duct regression in females, as was very apparent in males. Nor did we find a specific right duct mesenchymal population linked to regression as in males. This implies that the mechanism of duct regression may be fundamentally different in the two sexes. Both males and female ducts express AMHRII and the required ALK2/3 type I receptors (S10A and S10B Fig). Hence, it is highly likely that AMH is responsible for right Müllerian duct regression in the female chicken embryo as in male embryos. We did detect less cell proliferation in the right female duct (S8D Fig), though we did not detect any elevated apoptosis markers. Deeper sequencing may have revealed a developmental program for right duct regression, but the current data indicates that regression occurs differently to that in males and requires further study.

The left female duct in the chicken embryo does not normally regress, despite the presence of AMH, and is considered to be protected by local estrogens. Estrogen is synthesised by female gonads in the chicken embryo, while little is synthesised by the male gonads [98–102]. Estrogen can block the duct regressing-activity of AMH [103,104] and there is some evidence for more estrogen receptors in the left than the right duct [105]. However, we detected *ESR1* (encoding Estrogen Receptor Alpha) in both left and right ducts during right duct regression at E11.0 (HH stage 37), not asymmetrically. Furthermore, we found that AMHRII is downregulated in the left female duct (S10B Fig), suggesting that asymmetric AMH sensitivity, rather than estrogen, may underlie the left-right difference in female ducts.

In summary, the study defines the molecular and cellular events that underpin Müllerian duct sexual differentiation in the avian model. scRNAseq reveals greater cellular complexity than previously appreciated, with both the MDE and MDM comprising heterogeneous cell populations. We define molecular signatures for MDE and MDM development, and for the unique regressing male duct mesenchyme. We find that the differentiating left female duct comprises transcriptionally distinct populations of cells along its cranio-caudal axis, pre-patterning the duct prior to post-embryonic morphological differentiation. We also find that bilateral male duct regression follows a different cellular and molecular pathway than the unilateral duct regression observed in females. While duct regression is thought to be mediated by gonadal-derived AMH in both sexes, the molecular pathways of regression must differ. This intriguing finding requires further analysis. Lastly, we identify the WNT pathway inhibitor, *DKK4*, as a player in chicken Müllerian duct regression. This study enhances our knowledge of Müllerian duct biology and provides a foundation for understanding Müllerian duct disorders.

## Methods

### Genetic sexing, histology and immunofluorescence

All research utilizing chicken embryonic tissue observed the ethical guidelines provided by Monash University and the National Health and Medical Research Council. Fertilized eggs were obtained from HyLine Brown chickens (*Gallus gallus domesticus*) from Specialised Breeders Australia, Huntly, Victoria. Fertilized eggs from Specific Pathogen-Free (SPF) chickens were provided by Australian SPF Services. The eggs were incubated under humid conditions at 37.5°C until they reached appropriate stages. Harvested embryos were sexed by PCR, in which ZW females were identified by the presence of a female-specific (W-linked) XhoI repeat sequence in addition to a 18S ribosomal gene internal control. ZZ males showed the 18S band only [106].

Urogenital systems were harvested every day from E6.5 (HH stage 30) to E11.5 (HH stage 37), fixed in Bouin’s Solution overnight at room temperature, dehydrated in 70% ethanol, and the Müllerian ducts were imaged in brightfield. At the Monash Histology Platform, the urogenital systems were processed in a tissue processor and embedded into paraffin. Transverse sections were cut on the microtome, collected onto Superfrost slides, stained with Haematoxylin and Eosin, and mounted with Aquamount. Images were taken on a Zeiss Axio Imager.A1 microscope and Zeiss AxioCam MRc 5 camera.

Urogenital systems were harvested and fixed for 12 minutes in 4% PFA/PBS, cryo-protected in 30% sucrose/PBS overnight at 4°C. Tissues were embedded in OCT and stored at -80°C. Transverse sections were cut on a cryostat and mounted onto Superfrost Plus slides. Sections were treated with 1% Triton X-100/PBS for 10 min at room temperature, washed in PBS and blocked in 2% BSA/PBS for 1 hour at room temperature. Sections were incubated overnight at 4°C with a rabbit anti-SOX6 primary antibody diluted 1:200 in 1% BSA/PBS. Following three washes in PBS, sections were incubated with a Alexa-Fluor 488-conjugated donkey anti-Rabbit secondary antibody diluted 1:2000 in 1% BSA/PBS for 1 hour at room temperature (see Key Resources Table for antibody information). Sections were counterstained with DAPI, mounted with Fluorosave, and images were taken on a Zeiss Axio Imager.A1 microscope and Zeiss AxioCam MRc 5 camera.

### Single cell RNA-sequencing and bioinformatics analysis

This study conducted single cell RNA sequencing (scRNAseq) analysis on Müllerian ducts at key phases of duct development. Fertile chicken eggs were incubated at 37.5°C such that different developmental stages were reached on the same day. On the day of harvest, Müllerian ducts were dissected from embryos at embryonic day E6.5 (HH stage 30), E8.5 (HH stage 35) and E11.0 (HH stage 37). Five samples were obtained for single-cell RNA sequencing. The first sample consisted of both male and female ducts at E6.5, corresponding to duct formation in both sexes. The next sample consisted of male ducts at E8.5, corresponding to the onset of duct regression in the male. The third sample consisted of female ducts at E8.5. The fourth sample consisted of only the left ducts of the E11.0 females, corresponding to duct differentiation, while the fifth sample consisted of only the right ducts, corresponding to female duct regression. The anterior tips of the right female duct, the region in which female duct regression is initiated, were also dissected and processed with the E11.0 right whole duct sample.

Each of the samples above included three tubes (technical replicates), except for the E11.0 Female right duct sample which included four tubes (three technical replicates for the whole duct and one technical replicate for the duct tips). Each technical replicate included at least three ducts or duct pairs (biological replicates). All tissues were dissected into PBS at room temperature until dissociation. The tissues were dissociated by adding 300µL of 0.25% Trypsin-EDTA to each tube, and incubating at 37°C for 18 minutes, shaking at 750RPM. The digestion was stopped with 75µL of neat Fetal Calf Serum (FCS). The dissociated cells were filtered by loading onto 35µm Cell Strainer FACS tubes. The strainer was washed twice by adding 200µL 10% FCS in PBS, and centrifuging at 600G, room temperature, for 1 minute. The cells were pelleted by spinning at 600G, room temperature for 3 minutes. The 10% FCS in PBS was removed, and the cells were resuspended in 1mL 0.04% BSA in PBS. The cells were spun at 400G, room temperature, for 5 minutes.

Next, each technical replicate was labelled with a molecular tag sourced from 10X Genomics, the Cell Multiplexing Oligos (CMOs), which enabled the three technical replicates for each sample to be run in a single microfluidics channel. The technical replicates can then be demultiplexed during bioinformatics analysis. The following CMOs were used to label three technical replicates for each sample: E6.5 Mixed Sex (CMO 304–306), E8.5 Female (CMO 307–309), E8.5 Male (CMO 310–312), E11.0 Female left duct (CMO 301–303), E11.0 Female right duct (CMO 304–306 for whole ducts, CMO 307 for duct tips). The CMO labelling protocol was carried out in accordance with the manufacturer’s instructions (10X Genomics document CG000391 Revision B). Each technical replicate was resuspended in 100µL CMO and incubated for 5 mins at room temperature. Following CMO labelling, the dissociated cells were washed three times in 1% BSA in PBS, on ice. Each technical replicate was diluted to 1000 cells/µL and determined to be of >95% viability using trypan blue staining and the Countess 3 Cell Counter. Each technical replicate was pooled in equal proportions into a single sample.

The samples were then submitted to the Monash Bioinformatics and Genomics Platform Genomics Node (Monash University) for single-cell RNA sequencing. For each sample, 16,500 cells at 1000 cells/µL were loaded to obtain a targeted recovery of 10,000 cells post-sequencing. Single-cell analysis was carried out using the 10X Genomics Chromium Controller and Chromium Single Cell 3′ Library & Gel Bead Kit V3.1, as per the manufacturer’s instructions (10X Genomics document CG000388 Revision D). Samples were subjected to 11 cycles of PCR for cDNA amplification and 13/14 cycles for library amplification. Completed libraries were pooled in an equimolar ratio along with a 5% PhilX Control Library V3, per the manufacturer’s instructions (Illumina document 15048776 V11). The prepared libraries were sequenced using an Illumina Novaseq at Azenta Life Sciences, using SBS chemistry, in paired-end (PE)-150 configuration, targeting 20,000 read pairs per cell for gene expression libraries and 5000 read pairs per cell for Cell Multiplexing libraries.

The raw data was processed using Cellranger Multi (Cellranger version 8.0.1), and reads were aligned against the Ensembl Gallus gallus reference genome (Ensembl release 112 Gallus_gallus.bGalGal1.mat.broiler.GRCg7b). Each CMO-labelled technical replicate was demultiplexed in Cellranger and loaded into R for removal of outlier cells and doublets. Doublets were identified using the scDblFinder package, and outliers were identified using the isOutlier function from the scran package. Subsequently, the dataset was processed through the standard Seurat analysis pipeline. Using the R package Seurat v5 [107], gene expression levels were normalized against the number of reads per cell. 15 principal components were used for dimensionality reduction, and the data was visualised in a two-dimensional Uniform Manifold Approximation and Projection plot (UMAP). Clustering was carried out at a Seurat resolution of 0.8. In the Loupe Browser v9.0.0 (10X Genomics), cluster markers were determined by differential gene expression analysis between selected clusters or against the whole dataset, using the negative binomial test [108]. The same method was used to carry out multi-sample comparisons (pseudobulk). Differential gene expression analysis was used to annotate cell identity, to exclude clusters of non-Müllerian duct origin from further analysis and assign the remaining clusters to known compartments of the Müllerian duct. In the Loupe Browser, the top differentially expressed genes for each cluster was hierarchically clustered and visualised as heatmaps coloured by gene Log2 fold-change. An interactive web application was generated with the Shinycell package in R, and was used to plot cell information and gene expression plots. Bubble plots of selected cluster markers were also generated in the Shinycell application, in which normalised gene expression was averaged, log-transformed and plotted. Cell cycle states were assigned to each cell using the CellCycleScoring score function from Seurat and the chicken orthologs of the cc.genes.updated.2019 set of genes, which included 52 G2M genes and 43 S genes. For assigning sex for the E6.5 timepoint, all the annotated genes on the W chromosome were used for a gene signature score calculation using the AddModuleScore_UCell from the UCell R package. E6.5 cells with a value >0.0002703 were assigned as female and <0.0002703 as male. This threshold was the 3rd Quartile value for the E8.5 male population and less than the 1st Quartile score for the E8.5 female distribution. Lastly, Pseudotime analysis was carried out in Python using the Palantir algorithm. The single-cell RNA seq data is publicly available on the NCBI Gene Expression Omnibus (GEO Accession GSE323377).

### Whole mount in situ hybridization

Whole mount in situ hybridization for spatial mRNA expression was carried out to identify novel cell populations identified by single-cell RNA sequencing, as well as assess the effects of *DKK4* and *AMH* over-expression. Urogenital systems were dissected from staged chicken embryos over E6.5 – 10.5 and fixed overnight in 4% paraformaldehyde in DEPC-PBS. Tissues were dehydrated using a graded methanol series in PBTX (PBS + 0.1% Triton X-1000), including 15-minute washes of 25%, 50%, 75%, 100% and 100% methanol. Tissues were then rehydrated to PBTX using the same methanol series and permeabilized in proteinase K 10μg/mL for up to 2 hours, depending on tissue size and embryo stage. Tissues were then washed in PBTX, re-fixed in glutaraldehyde/PFA/PBTX for 20 minutes and placed into pre-hybridization solution overnight at 65°C (50% formamide, 5 x SSC, 0.1% Triton X-100, 0.5% CHAPS, 5mM EDTA, 50μg/mL Heparin, 1mg/mL yeast RNA, 2% blocking powder). Riboprobe templates were amplified from gonadal and whole embryo cDNA using gene specific primers. The T3 and T7 RNA polymerase promoter sites were included in the tails of the primers to enable the synthesis of RNA probes via *in vitro* transcription. Antisense RNA probes were synthesized using T3 RNA polymerase and the DIG-labeling kit (Roche, #11277073910) which labels probes with Digoxygenin. Sense probes synthesized using the T7 RNA polymerase served as negative controls. Precipitated probes were added to pre-hybridized tissues at 10μL per tube, and hybridization was carried out overnight at 65°C. Two stringency washes were carried out at 65°C for 30 minutes (2x SSC; 0.1% CHAPS x 30 minutes; 0.2x SSC; 0.1% CHAPS), followed by equilibration in TBTX (50mM Tris-HCl, pH 7.5, 150mM NaCl, 0.1% Triton X-100). Tissues were then blocked (1x TBTX; 2% BSA, 10% Sheep serum) and then treated overnight with anti-DIG antibody conjugated with alkaline phosphatase. Following five 1-hour washes in 0.2% BSA in TBTX at room temperature, tissues were equilibrated in NTMT (100mM NaCl, 100mM Tris-HCl, pH9.5, 50mM MgCl2, 0.1% Tween-20). Tissues were then incubated in color reaction (340μg/mL NBT and 175 μg/mL BCIP in NTMT) at room temperature until specific staining developed, between 1–4 hours. Following imaging of whole mounts on a Leica M165 FC Fluorescent Stereo Microscope and Leica MC190 HD camera, tissues were over-stained in the BCIP/NBT colour reaction by rocking for 2 days at room temperature, and cryo-protected in sucrose. Tissues were embedded in OCT compound and frozen at -80°C. Transverse sections were cut on a cryostat at 8µm and mounted onto SuperFrost Plus slides using Aquamount. Images were taken on a Zeiss Axio Imager.A1 microscope and Zeiss AxioCam MRc 5 camera.

### *AMH* and *DKK4* over-expression

To generate the AMH overexpression construct, emGFP-T2A-cAMH was cloned into the RCASBP(A) vector using the Gibson Assembly Cloning kit (New England Biolabs), as previously described [109]. This is a method for inducing ubiquitous gene expression using the avian retroviral vector, Replication-Competent Avian sarcoma-leukosis virus, with a Splice acceptor, Bryan RSV Pol (RCASBP). High quality plasmid DNA (RCASBP(A)-emGFP-T2A-FLAG or RCASBP(A)-emGFP-T2A-cAMH) was prepared using the NucleoSpin plasmid maxiprep kit (Macherey-Nagel). The vector and insert were confirmed by Sanger sequencing. The DNA was then transfected into DF-1 cells, a chicken embryonic fibroblast cell line, using Lipofectamine 2000 (Life Technologies), and the virus was harvested as previously reported [109]. Briefly, the DF-1 cells were passaged into six T-175 flasks and grown until super-confluent. The cell media was replaced with 1% FCS DMEM and harvested over 6 consecutive days, from which the virus was purified and concentrated by ultracentrifugation. To induce global expression of *AMH*, the purified RCASBP(A)-emGFP-T2A-cAMH virus was injected into unincubated blastoderms *in ovo*. Sterile Fast Green tracking dye was added to an aliquot of purified RCASBP(A)-emGFP-T2A-cAMH virus to a final concentration of 0.025%, and approximately 1–2µL was injected into the blastoderm. An RCASBP(A)-emGFP-T2A-FLAG construct was used as a control. Following the blastoderm injection, eggs were sealed with clear tape and incubation resumed until E9.5. Subsequently, GFP expression and whole-mount *in-situ* hybridisation for *AMH* mRNA was used to confirm the over-expression of *AMH* in the urogenital system (S11 Fig).

To induce over-expression of *DKK4* in the left Müllerian duct, *in ovo* electroporation of chicken embryos at E2.5 (HH stage 14) was performed as previously described [54]. This method allows delivery of exogenous genes into the coelomic epithelium, in the region of the presumptive Müllerian duct. CAGS→GFP-T2A-DKK4 was cloned into a TOL2 based vector. In the presence of co-electroporated plasmid expressing transposase enzyme (p-CAGS-Transposase), the CAGS→GFP or CAGS→GFP-T2A-DKK4 cassette is stably integrated in into the genome, permanently expressing GFP (control) or GFP and the gene of interest [110,111]. High-quality plasmid DNA for pTOL2(CAGS→GFP) or pTOL2(CAGS→GFP-T2A-DKK4) was prepared using the NucleoSpin plasmid maxiprep kit (Macherey-Nagel). The electroporation mix comprised of pTOL2(CAGS→GFP) or pTOL2(CAGS-GFP-T2A-DKK4) at 1μg/uL, p-CAGS–Transposase at 0.6 μg/uL, 0.16% carboxy methylcellulose, 1 mM MgCl2, and 1.3% Fast Green tracking dye in PBS. Chicken eggs were opened at E2.5 (HH stage 14–15), visualized with Indian Ink/PBS injected under the embryo and electroporation mix was injected into the left coelom using fine glass capillary needles. Within 60s of injection, insulated electrodes were placed sub-laterally and dorso-medially to the embryo. Current was applied using a TBX Electro Square Electroporator. Electroporation conditions were: 40V; 2 pulses; 10msec width; 150msec space between pulses [54]. After electroporation, eggs were sealed with clear tape and incubation resumed until E9.5. Subsequently, GFP expression and whole-mount *in-situ* hybridisation for *DKK4* mRNA was used to confirm over-expression of *DKK4* in the left Müllerian duct.

### Müllerian duct area and WNT4 staining quantification in tissue sections

To assess Müllerian duct area and WNT4 WISH staining intensity following *DKK4* overexpression, three GFP control and three *DKK4* overexpression embryos were used. For each embryo, 10μm transverse sections were obtained from the posterior section of the Müllerian ducts using a cryostat. For each embryo, three transverse sections were imaged on a Zeiss Axio Imager.A1 microscope and Zeiss AxioCam MRc 5 camera. Image analysis was conducted in Fiji, in which the cross-sectional area of the whole duct, duct epithelium (MDE) and mesenchyme (MDM) were measured by manual selection. The units of measurement were converted from px^2^ to μm^2^ by measuring a scale bar under the same magnification. To quantify WNT4 staining in the mesenchyme, the images were inverted, converted to 8-bit format, and mean pixel intensity was measured. The background pixel intensity of each image was similarly measured and subtracted from the WNT4 staining values. In Graphpad Prism 11, nested t-tests were performed between the control and *DKK4* overexpression groups, to compare the cross-sectional area of the whole duct, MDE, and MDM, as well as the WNT4 staining intensity in the MDM. Bar-and-scatter plots were generated in Graphpad Prism 11.

## Supporting information

S1 Table Differential gene expression analysis, cell barcode and sample information. (XLSX)

S2 Table PCR oligonucleotide primers used for in situ hybridisation probe synthesis and cloning. (DOCX)

S1 Fig Müllerian duct sexual differentiation over time. **(A)** Whole mount urogenital systems from E6.5 (stage 30) to E11.5 (stage 37). Müllerian ducts are denoted by the dotted lines. At E6.5 (stage 30), male and female ducts are morphologically identical. In males, bilateral duct regression occurs in a caudo-cranial direction from E8-E11.5 (stage 32–37) (arrow #1). In females, from E7.5-8.5 (stage 32–34), both ducts thicken. From E9.5-E11.5 (stage 35–37), the left female duct continues to increase in size while the right female duct regresses in a cranio-caudal direction (arrow #2). Scale bar = 1mm. **(B)** Mid-level transverse sections of the Müllerian duct from E6.5 (stage 30) to E11.5 (stage 37), stained with Haematoxylin and Eosin. Ducts are morphologically identical at E6.5 (stage 30). By E8.5 (stage 34), sexual dimorphism has emerged; female ducts increase in size, while male ducts are regressing. This dimorphism is more pronounced at E11.5 (stage 37). MDM = Müllerian Duct Mesenchyme. MSE = Müllerian Surface Epithelium. MDE = Müllerian Duct Epithelium. WD = Wolffian duct. Ms = Mesonephros. Scale bar = 25µm.
(TIF)

S2 Fig Differential gene expression analysis to identify non-Müllerian duct cells and Müllerian duct cells. **(A)** Bubble-plot of selected marker genes identified six clusters as non-Müllerian duct cells. Clusters 12 and 22 were identified as erythrocytes, Cluster 13 as Wolffian duct, Cluster 19 as immune cells, Cluster 20 as endothelial cells. Cluster 21 lacked defining differentially expressed transcripts but expressed high levels of ribosomal and mitochondrial genes, suggesting apoptotic cells of varied origin. **(B) N**on-Müllerian duct cells represented on a UMAP, relative to Müllerian duct cells (grey). **(C)** Heatmap of significantly upregulated and downregulated genes in each Müllerian duct cell cluster.
(TIF)

S3 Fig Cell cycle analysis. **(A)** Cell phase analysis showing clusters in the G1 phase (pink), S phase (blue) and G2M phase (green). One proliferative cluster was identified in the Müllerian Duct Epithelium (MDE), and in the Müllerian Surface Epithelium (MSE). One proliferative cluster was also identified in both the female and male Müllerian duct mesenchyme (MDM). **(B)** PCA of cell cycle genes, in which each cell was assigned a cell cycle state based off expression of 52 G2M genes and 43 S genes from Seurat. **(C)** Expression of 95 genes was used to determine cell cycle states, and 8 genes are shown here.
(TIF)

S4 Fig Transcriptomic profile of the E6.5 mixed sex sample. **(A)** Co-expression plot of the female marker HINTW (pink) and the housekeeping gene GAPDH (blue) on the global UMAP, showing that male and female cells in this sample localise to the same clusters. **(B)** UMAP of E6.5 cells only, showing that male and female cells localise to the same clusters. (**C)** Heatmap of the top 25 differentially expressed genes in MSE Clusters 4 and 16, colour coded by gene expression level (Log2 Fold Change), used to identify novel candidate genes involved in duct formation. **(D)** Gene expression plots of MSE-enriched genes at E6.5/stage 30. **(E)** Validation of SOX6 expression in the MSE of an E6.5 female, by immunofluorescence (arrowheads). MDE = Müllerian Duct Epithelium. MDM = Müllerian Duct Mesenchyme.
(TIF)

S5 Fig Transcriptomic profile of the E6.5 mixed sex Müllerian duct mesenchyme (MDM). **(A)** Transcriptional clustering identified one mesenchymal cluster enriched in this sample, Cluster 17 (circle), enriched for known markers such as *WNT4*. Mesenchymal clusters 3 and 10 were present in this sample and in all other samples (circle). **(B)** Epithelial-to-mesenchymal transition (EMT) genes were enriched in the mesenchyme at E6.5. **(C)** Novel markers such as *FOXE1* were identified in the E6.5 mesenchyme. **(D)** Clusters 3 and 10 are mesenchymal clusters that are present in all samples and are enriched for *ADGRA1* and *EBF1* expression. *ADGRA1 in situ* hybridisation indicates that *ADGRA1* is expressed in the mesonephros (Ms) at E6.5, and subsequently in the duct mesenchyme (MDM) adjacent to the mesonephros at E8.5. EBF1 is also present in the MDM adjacent to the Ms at E11.0. Scale bar = 25µm.
(TIF)

S6 Fig Transcriptomic profile of the E6.5 (stage 30) mixed sex Müllerian duct epithelium (MDE). **(A)** At E6.5, the male and female MDE have similar transcriptomic profiles, except for the female marker HINTW. At E8.5, the MDE shows sexually dimorphic gene expression. **(B)** Heatmap of the top differentially expressed genes in each MDE cluster, compared against the other MDE clusters [8,14,15]. GFRA1 is a marker of cluster 8 while MSX2 is a marker of cluster 15 (Black boxes). Whole mount *in situ* hybridisation shows that *GFRA1* is expressed along the length of the MDE excluding the posterior pole from E6.5 to E11.0. *MSX2* is expressed in the posterior pole of the MDE in the E8.5 and E11.0 female. *MSX1* is expressed in the posterior pole of the MDE in the E11.0 female. Scale bar = 25µm.
(TIF)

S7 Fig Transcriptomic profile of the E8.5 (stage 34) female Müllerian duct mesenchyme (MDM). **(A)** Three MDM clusters were enriched in this sample, Clusters 1, 6 and 11. MDM Clusters 0, 2 and 5 are present in smaller proportions (dotted line). The heatmap shows top differentially expressed genes in Clusters 1, 6 and 11. **(B)** Known markers of the MDM were enriched in cluster 1, 6 and 11. **(C)** Cluster 6 consisted of proliferating cells marked by *CENPF* expression. **(D)** Cluster 11 is marked by intermediate levels of gene expression between Clusters 1 and 6. **(E)** Clusters 2 and 5 are enriched for ADAMTS6 expression while cluster 0 is enriched for *CDH13* expression.
(TIF)

S8 Fig Comparison of the Müllerian duct mesenchyme (MDM) in the left and right ducts of the E11.0 (stage 37) female. **(A)** The left duct, right duct and right duct anterior tips consisted of the same MDM clusters, clusters 0, 1. 2, 5, 6 and 11 (dotted line). **(B)** Shared transcripts are expressed in the left ducts, right ducts, and right duct anterior tips. **(C)** *HOXC10* is enriched in Cluster 2 and 5 in the left duct but absent from cluster 2 and 5 in the right anterior duct tips. **(D)** Significantly lower (FDR < 0.05) proportion of proliferating cluster 6 in the right duct compared to the left duct.
(TIF)

S9 Fig Segmentally expressed genes along the anterior-posterior axis of the Müllerian duct mesenchyme. *HOXA9* and *HOXA10* are enriched throughout the Müllerian duct mesenchyme from E6.5 (stage 30) to E11.0 (stage 37). From E8.5, *HOXA11* expression is restricted to the top left of the UMAP (arrow). From E8.5, *HOXA13* is expressed in a smaller subset of cells in the top left of the UMAP compared to *HOXA11*. *SRD5A2*, a novel transcript identified in the posterior region of the female duct, also shows restricted expression to the top left of the UMAP at E8.5 and E11.0. Top-left localisation of cells on the UMAP corresponds to posterior localisation on the Müllerian duct (arrows).
(TIF)

S10 Fig Genes associated with male Müllerian duct regression at E8.5 (stage 34). **(A)** Receptors of AMH signalling, *AMHR2* and *ATVR1,* are expressed in all compartments of the Müllerian duct, including the mesenchymal clusters associated duct regression (Clusters 7, 9 and 18). **(B)** Whole-mount *in situ* hybridisation shows that *AMHR2* mRNA is expressed in the coelomic epithelium (CE) of the presumptive Müllerian duct at E4.5, and in the duct mesenchyme (MDM) at E6.5, in both males and females. In the male, at E8.5 and E10.5, *AMHR2* is expressed in the mesenchyme of the regressing duct. In females, at E10.5, AMHR2 is expressed in the right regressing duct but is absent from the left duct (dotted line). Scale bar = 50µm. **(C)** Top differentially expressed genes (DEGs) in each male MDM cluster.
(TIF)

S11 Fig *AMH* mRNA expression and gonad morphology at E9.5 (stage 35) following *AMH* over-expression using a viral vector. In the *GFP* controls, female gonads (G, dotted lines) are asymmetrical and weakly express *AMH*, while male gonads are symmetrical and strongly express *AMH*. In *AMH* overexpression tissues, both male and female gonads are thin and symmetrical, and strongly express *AMH. AMH* is also expressed in the mesonephric kidney (Ms). Scale bar = 50µm.
(TIF)

S12 Fig *WNT4* mRNA expression and duct morphology at E9.5 (stage 35) following *DKK4* over-expression in the left Müllerian duct. In the *GFP* controls, both left and right female ducts are thick and express *WNT4* throughout the duct mesenchyme (MDM). In the male, the regressing ducts are thin and *WNT4* is weakly expressed in the MDM. Following *DKK4* overexpression of the left female duct, *WNT4* is weakly expressed in the MDM, and the duct is thin (arrow) due to a loss of mesenchyme (arrowhead). In the male left duct, *WNT4* expression and duct morphology is unchanged following *DKK4* overexpression. MDE = Müllerian duct epithelium. Ms = Mesonephros. Scale bar = 50µm.
(TIF)

S13 Fig GFP and *DKK4* mRNA expression following *DKK4* overexpression in the left Müllerian duct of E9.5 (stage 35) females. **(A)** In GFP controls, the left duct is thick, and GFP is expressed along the length of the duct. Following *DKK4* overexpression, the duct is thin and wispy, and regression is more pronounced in the posterior region of the duct (arrows). **(B)** Whole-mount *in situ* hybridisation for *DKK4* mRNA. In the GFP controls, both ducts are thick and robust (dotted lines), and *DKK4* expression is absent. Following DKK4 overexpression in the left duct, the ducts are regressed (arrowheads), and the remaining tissue shows strong expression of *DKK4.* Scale bar = 1mm.
(TIF)

S14 Fig GFP and *DKK4* mRNA expression following *DKK4* overexpression in the Müllerian duct at E6.5 (stage 30). Following *DKK4* overexpression, the duct (MD) has formed along the length of the mesonephros (Ms). The morphology of the DKK4-overexpressing duct is comparable to that of the unelectroporated right duct and the GFP control, as indicated by the presence of a duct epithelium (MDE) and mesenchyme (MDM). Scale bar = 25 µm.
(TIF)
