# Supplementary material for: New insights into Müllerian duct differentiation provided by single cell transcriptomics in the chicken embryo
Source: PLoS Genet. 2026 Oct 9;22(10):e1012302. doi: 10.1371/journal.pgen.1012302 (PMC13652249; doi:10.1371/journal.pgen.1012302)
Supplement: Supplementary file 1 [file pgen.1012302.s002.docx]

# **Supplementary Table 2. Primers for *in situ* hybridisation probe synthesis and cloning**

| Function | Oligonucleotide name | Sequence |
| --- | --- | --- |
| Cloning  (*DKK4* overexpression construct) | *DKK4*  Forward Primer | GGAAAATCCCGGCCCCATGCGGGCTCTGCTGGCG |
| Cloning  (*DKK4* overexpression construct) | *DKK4*  Reverse Primer | CATCGTCGTCCTTGTAGTCACCTCAGTGCCGCTGGCAAGTGTGCAGGCGG |
| Probe Synthesis | *MSX1*-T7 Forward Primer | GTAATACGACTCACTATAGGGGAGAGCGACAAACCCAAGGT |
| Probe Synthesis | *MSX1-*T3 Reverse Primer | CAATTAACCCTCACTAAAGGGGCTCGGCCCTATGTAAGGTG |
| Probe Synthesis | *MSX2*-T7 Forward Primer | GTAATACGACTCACTATAGGGACAGGAGTTCGCGTTAGCAA |
| Probe Synthesis | *MSX2-*T3 Reverse Primer | CAATTAACCCTCACTAAAGGGAAGGAGAATACCGGGGACGA |
| Probe Synthesis | *SRD5A2*-T7 Forward Primer | GTAATACGACTCACTATAGGGCCGCCGCCGCTCTAATAA |
| Probe Synthesis | *SRD5A2*-T3 Reverse Primer | CAATTAACCCTCACTAAAGGGAGGTAGCGATGGCAAAACCA |
| Probe Synthesis | *EBF1*-T7 Forward Primer | GTAATACGACTCACTATAGGGTCGGAAGGTACGCCTTCTTATC |
| Probe Synthesis | *EBF1*-T3 Reverse Primer | CAATTAACCCTCACTAAAGGGGACCATGTTGGCTGGTGAGA |
| Probe Synthesis | *ESR1*-T7 Forward Primer | GTAATACGACTCACTATAGGGAGCCTGGCAGGATTTCACTC |
| Probe Synthesis | *ESR1*-T3 Reverse Primer | CAATTAACCCTCACTAAAGGGAGAGGTCGTAGAGCGGAACT |
| Probe Synthesis | *CPXM2*-T7 Forward Primer | GTAATACGACTCACTATAGGGGCAAGGATTGGTTCGAGCTG |
| Probe Synthesis | *CPXM2*-T3 Reverse Primer | CAATTAACCCTCACTAAAGGGTCCAGCTCTCAGAACGACAAG |
| Probe Synthesis | *GFRA1*-T7 Forward Primer | GTAATACGACTCACTATAGGGCACCAGCCACATATCCTCGG |
| Probe Synthesis | *GFRA1*-T3 Reverse Primer | CAATTAACCCTCACTAAAGGGGAAGCACAGAAGACCGGTGA |
| Probe Synthesis | *AMHR2*-T7 Forward Primer | GTAATACGACTCACTATAGGGCGGTCGCCATAAAAGCCTTCGC |
| Probe Synthesis | *AMHR2*-T3 Reverse Primer | CAATTAACCCTCACTAAAGGAGAAACTGCTCCGGAACCTCC |
| Probe Synthesis | *DKK4*-T7 Forward Primer | GTAATACGACTCACTATAGGGATGCGGGCTCTGCTGGC |
| Probe Synthesis | *DKK4*-T3 Reverse Primer | CAATTAACCCTCACTAAAGGGTCAGTGCCGCTGGCAAGTGTG |
| Probe Synthesis | *LVRN*- T7 Forward Primer | GTAATACGACTCACTATAGGGTCCATGACCCAAGCTACGTG |
| Probe Synthesis | *LVRN*-T3 Reverse Primer | CAATTAACCCTCACTAAAGGAGGGCTGCGATGATCCATTT |
| Probe Synthesis | *PLK2*-T7 Forward Primer | GTAATACGACTCACTATAGGGTGCTCGGAAAGGGTGGATTC |
| Probe Synthesis | *PLK2*-T3 Reverse Primer | CAATTAACCCTCACTAAAGGGACTAGGCCGGTCTTCAGGAT |
| Probe Synthesis | *ADGRA1*-T7 Forward Primer | GTAATACGACTCACTATAGGGTGGGAAACGTAGGACGGTTG |
| Probe Synthesis | *ADGRA1*-T3 Reverse Primer | CAATTAACCCTCACTAAAGGGGACTAGGCTCCCATGCCATC |
